# Integrated multi-omics and WGCNA analyses reveal pathways and candidate genes associated with branching flower development in *Nymphaea prolifera*: focusing on hormone homeostasis and flavonoid biosynthesis

**DOI:** 10.1186/s12870-026-09140-2

**Published:** 2026-05-29

**Authors:** Yuwei Tang, Jiahui Li, Miaoqin Wei, Zhenjun Bin, Zuzheng Lu, Jiahui Zhao, Xiaoyu Gao, Qun Su

**Affiliations:** 1 Guangxi Subtropical Crops Research Institute, Nanning, 530001 China; 2 Flowers Research Institute, Guangxi Academy of Agricultural Sciences, Nanning, Guangxi 530007 China; 3 Nanning Qingxiushan Scenic and Historic Tourism Development Co., Ltd., Nanning Botanical Garden, Nanning, 530029 China

**Keywords:** *Nymphaea prolifera*, Branching flower, Weighted gene co-expression network analysis, Endogenous hormone, Flavonoid biosynthesis

## Abstract

**Background:**

*Nymphaea prolifera* employs a unique asexual reproductive strategy in which its normal flowers transform into branching flowers. These are specialized structures that develop vegetative propagules. These propagules can directly sprout into new plants. As the branching flowers mature, they detach from the parent plant and form new, independent individuals. However, the underlying molecular mechanisms that govern this distinctive reproductive phenomenon remain unclear.

**Results:**

In this study, microscopic observations were conducted on normal flowers and branching flowers of *Nymphaea prolifera*. The results showed that reproductive organ tissues such as the pistils (including ovaries and ovules) of branching flowers were completely transdifferentiated into vegetative propagules, which could continuously generate multiple generations of branching flowers. Analyses of endogenous hormones, transcriptomes, and metabolomes were performed on branching flowers at four developmental stages (bud stage, early flowering stage, middle flowering stage, and late flowering stage) as well as on normal flowers. The results revealed significant differences in eight hormone compounds between branching flowers and normal flowers, including 2 cytokinins, 4 auxin-related compounds, ABA, and GA₄. GO and KEGG functional enrichment analyses indicated that both differentially expressed genes (DEGs) and differentially accumulated metabolites (DAMs) were significantly enriched in the flavonoid biosynthesis pathway. Furthermore, the expression levels of genes and metabolites associated with this pathway were gradually up-regulated during the development of branching flowers. Weighted gene co-expression network analysis (WGCNA) identified three core modules (MEgreen, MEblue, MEturquoise) that were highly correlated with hormone and flavonoid metabolism, and screened out potential candidate genes *IAA17*,* ARF6*,* ABF1* that may be associated with endogenous hormone and flavonoid metabolism.

**Conclusions:**

This study reveals the morphological and molecular characteristics of branching flower development in *N. prolifera*. It identifies the hub genes, modules and metabolic pathways involved in this phenomenon, thereby providing a comprehensive morphological and molecular basis for elucidating this species’ unique asexual reproductive mechanism.

**Supplementary Information:**

The online version contains supplementary material available at 10.1186/s12870-026-09140-2.

## Background

Water lilies (*Nymphaea*) are perennial aquatic herbs comprising 45–50 species in the family *Nymphaeaceae* distributed across five subgenera (*Anecphya*, *Brachyceras*, *Hydrocallis*, *Lotos*, and *Nymphaea*). They have high ornamental and cultural value and are native to temperate, subtropical, and tropical regions worldwide. These plants have extensive root systems and exhibit high nitrogen and phosphorus requirements during growth. Furthermore, they demonstrate a pronounced capacity for phytoremediation of aquatic environments by efficiently absorbing heavy metals such as mercury, chromium, and manganese [1]. Phylogenetically, the order *Nymphaeales*, which includes water lilies, along with *Amborellales* and *Austrobaileyales*, constitute the ANA grade of basal angiosperms. Consequently, water lilies serve as crucial model organisms for investigating the early evolution of floral traits [2].

*Nymphaea prolifera* (subgenus *Hydrocallis*), a neotropical species is native to Latin American countries, including Argentina, Brazil, and Ecuador. This species was originally described by Wiersema from Argentina [3, 4]. Subsequently, this plant was also discovered in Mato Grosso and Mato Grosso do Sul, Brazil [5], Paraguay [6], and it has been documented that *N. prolifera* can reproduce asexually via tuber-bearing flowers. The flowers of *N. prolifera* are capable of synthesizing and releasing specific benzene-related compounds, which act as crucial natural allelochemical defense substances in the flowers and exhibit significant repellent biological activity against aphids [7]. It exhibits the nocturnal anthesis characteristic of its subgenus. *N. prolifera* has a highly efficient asexual reproduction method, in which its flowers often mutate into branching flowers (known as parent flowers) whose internal vegetative propagules produce new leaves, flowers, and root systems. Crucially, these flowers may develop into conventional flowers or become the second generation of branching flowers (known as daughter flowers). Daughter flowers can continue to vegetative reproduce a third generation of branching flowers called grandchildren. When branching flowers develop to a certain stage, their pedicels rot and grow from the mother to new plants, while branching flowers will completely evolve into the tubers of new plants. Grob et al. found that the pistils of water lily flowers were replaced by vegetative propagules through histological observation, thus forming branching flowers with asexual reproduction ability [8]. This unusual phenomenon raises a unique biological question: why and how do reproductive organs undergo complete transdifferentiation into vegetative propagules in this specific species?

This process involves molecular transitions between vegetative and reproductive growth programs. Numerous reports have documented the transition from vegetative to reproductive growth in plants. For example, INDETERMINATE1 acts in coordination with *MYB31* and *TCP* family members to positively regulate floral transition through the autonomous pathway in temperate maize [9]. *HB34* regulates the transition from vegetative to reproductive growth by the *HB34-WRKY40-FT* transcriptional cascade in *Arabidopsi*s [10]. The AP2-family transcription factor BARE RECEPTACLE modulates floral organogenesis through auxin signaling pathways in woodland strawberry [11]. However, there are few reports of reproductive organs transforming into vegetative propagules in angiosperms; in particular, the phenomenon of pistils undergoing complete transdifferentiation to form vegetative propagules has not yet been thoroughly elucidated.

The branching of waterlily flowers represents vegetative propagation. The vegetative propagules are derived from the modification of reproductive organs (flowers pistil), which is unique to these species. In nature, there are bulbils, corms, and stem tubers that are similar to the branching flowers of water lilies, especially when the vegetative tissue attachment position is located on the inflorescence. Extensive studies have been conducted on vegetative reproduction. Plant hormones orchestrate bulbil initiation, growth, and maturation through complex species-specific signaling networks. In *Titanotrichum oldhamii*, the floral meristem undergoes a loss of its intrinsic identity, whereupon vegetative meristems develop in its immediate vicinity and give rise to vegetative bulbils within the original floral structure [12]. In *Agave tequilana*, reproductive failure or inadequate flower development stimulates the formation of vegetative bulbils at the bracteoles. *AtqPIN1* and *AtqSoPIN1* coordinate IAA transport to promote the initiation of bulbils, ensuring survival in a hostile environment [13]. Cytokinins (CTK) play a pivotal role in bulbil formation. *Triploid Lilium lancifolium* is completely sterile and thus incapable of sexual reproduction; instead, it compensates for this reproductive limitation by producing abundant bulbils, which enable efficient asexual propagation. These bulbils serve as the primary vegetative reproductive organs of this triploid species, and their development is well-documented to be positively regulated by CTKs [14]. Among the key regulators of this process, Type-B response regulators act as crucial mediators of primary CTK signaling, driving the expression of CTK-inducible genes to promote bulbil initiation [15]. 6-Benzylaminopurine (6-BA), a commonly used synthetic CTK, induces bulbil initiation in *L. lancifolium* by promoting cell proliferation [16]. GA_3_ moderately enhances bulbil production, whereas higher concentrations suppress bulbil formation [17]. These studies revealed the regulation of hormones in vegetative propagule development.

Plant tubers are often nutrient-rich. When the branching flowers of water lilies develop to a certain extent, they detach from their mothers. After which, vegetative propagules evolve into tubers that provide nutrients for the growth of new plants. Tubers are often rich in flavonoids and other compounds. For example, the bulbils and tubers of *Pinellia ternata* have a high total flavonoid content [18]. *Dioscorea cirrhosa* tubers are rich in flavonoids, in particular, epicatechin and proanthocyanin B2 are key metabolites that exhibit high levels [19]. During the early expansion of sweet potato root tubers, differential metabolites are mainly concentrated in amino sugar and nucleotide sugar metabolism and flavonoid biosynthesis [20].

Based on studies of vegetative reproduction in other plants, which endogenous hormones influence the formation of the unique “branching flower” phenomenon in *N. prolifera*? What role does the accumulation of flavonoids play in the development of vegetative propagules? These scientific questions remain unclear. In this study, we performed transcriptome sequencing and metabolomics assays on vegetative propagules of branching flowers and tissues from the corresponding pistil region in normal flowers of *N. prolifera* to assess differences in their expression at the metabolic and molecular levels. We screened for genes related to vegetative propagule formation using weighted gene co-expression network analysis (WGCNA). These findings contribute to a deeper understanding of metabolite formation and related gene changes in the vegetative propagules of branching flowers and provide insights into the formation of branching flowers.

## Materials and methods

### Plant material

Three-year-old *N. prolifera* were planted at Guangxi Subtropical Crops Research Institute in Nanning, Guangxi, China (108°20′E, 22°53′N). And cultivated under open-air natural environmental conditions without artificial greenhouse control. The cultivation site is a dedicated water lily germplasm resource nursery with unobstructed sunlight exposure, ensuring the plants received sufficient natural light throughout the growth period. Unified and standardized agronomic management measures were adopted during the whole growth cycle.

Plants were cultivated using the pot-in-pot method: a large pot with a top diameter of 90 cm and a height of 60 cm was filled with clean tap water, and a small pot with a top diameter of 30 cm and a height of 20 cm was filled with uniformly mixed pond mud (the mud was air-dried and sieved to remove gravel and plant residues, pH 6.5–7.0). To minimize the impact of environmental conditions, we collected all samples in June 2024 (the full flowering and asexual reproduction stage of *N. prolifera*), including normal and branching flowers, in different periods. During this period, the local average daily temperature was 25 to 31 ℃.

**Fig. 1:**
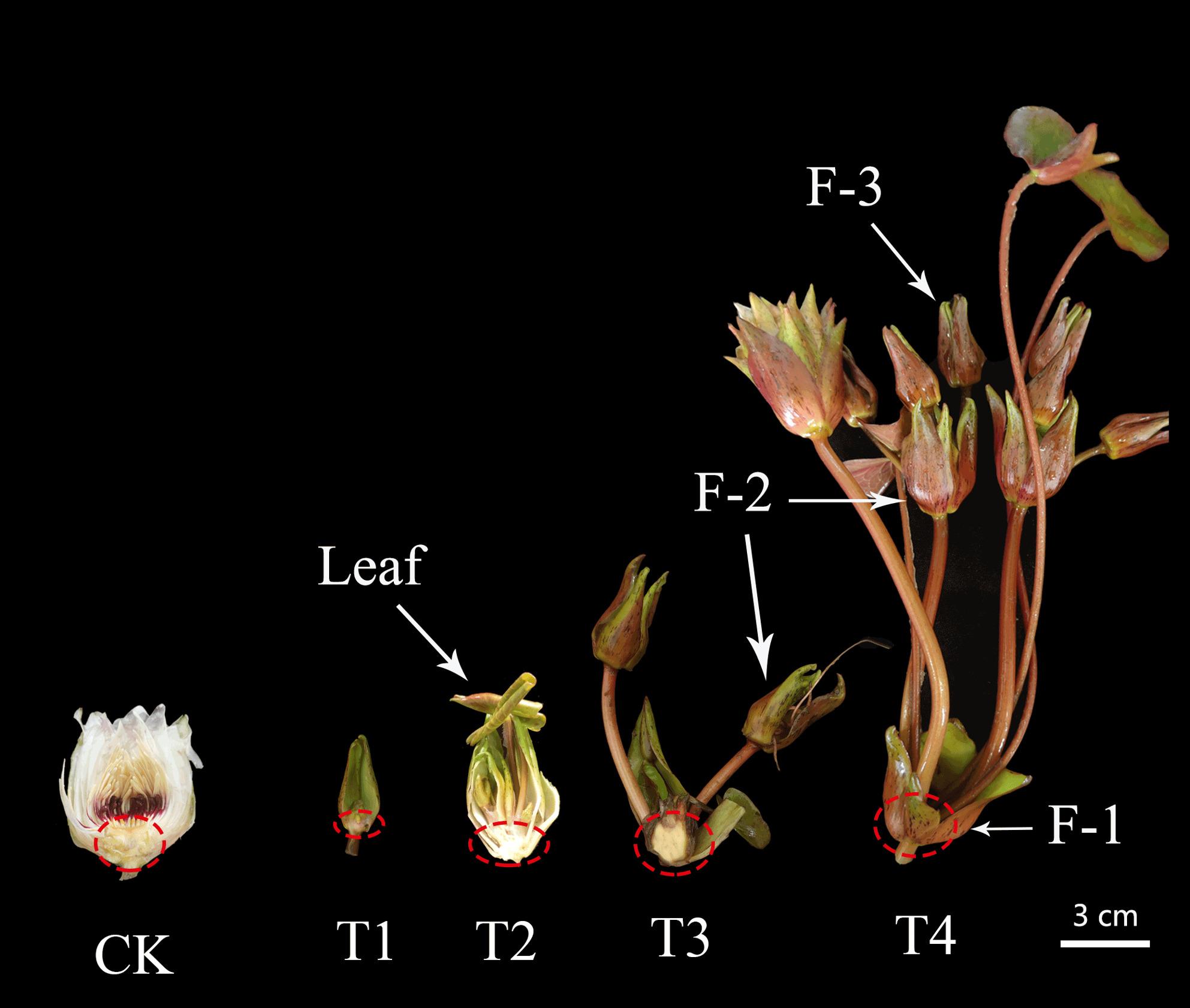
Phenotypes of *Nymphaea prolifera* with normal and branching flowers. Red dotted lines indicate collected samples. The scale is 3 cm. F-1: First generation of branching flower, also referred to as mother flower; F-2: Second generation of branching flower, also referred to as daughter flower; F-3: Third generation of branching flower, also referred to as granddaughter flower; Leaf: New leaves sprouting from the branching flower. CK: Normal flowers fully opened on the first day with the entire pistil tissue sampled as the control group. T1 (bud stage): Branching flower presents a flower bud morphology, with non-swollen vegetative propagules, no differentiated F-2 and no differentiated leaves; T2 (early flowering stage): Branching flower remains in a floral bud morphology with unopened sepals, the vegetative propagules are swollen, with differentiated F-2 at the bud stage inside and curled leaves; T3 (middle flowering stage): Branching flower blooms with opened sepals, the vegetative propagules are further swollen, with differentiated F-2 at the early flowering stage and expanded leaves; T4 (late flowering stage): Branching flower blooms with opened sepals, basically forming an independent plant morphology with elongated petioles, and differentiated F-2 at the middle flowering stage simultaneously

The test samples were divided into five groups. For the normal flowers fully opened on the first day, the entire pistil tissue was sampled and used as the control group (CK). Vegetative propagules were collected as samples from the branching flowers of water lily at four developmental stages (bud, early flowering, middle flowering, and late flowering), with the sampling positions strictly corresponding to those of the pistils in normal flowers. These samples were designated as treatment groups T1, T2, T3 and T4, respectively (Fig. 1). When collecting samples, immediately remove the sepals of the branching flower. Samples were frozen in liquid nitrogen immediately after collection for RNA extraction and metabolite analyses. LC-MS hormone detection, transcriptome sequencing, and qRT-PCR were performed for three biological replicates. Metabolite profiles are subject to substantial biological variation caused by individual differences, environmental fluctuations, and physiological status. Therefore, non-targeted metabolomics was performed using six biological replicates, and each replicate contained tissues pooled from at least three individual *Nymphaea prolifera* plants. In Fig. 1, samples T1 to T4 all represent branching flowers. The term “branching flower” refers to the entire structure containing these vegetative propagules. The regions marked by red circles indicate the vegetative propagules of the branching flower. After the branching flower detaches from the maternal plant and develops independently, these vegetative propagules will eventually differentiate into tubers of the new individual.

### Paraffin section examination and scanning electron microscopy

Vegetative propagules were selected from branching flowers as the experimental group, using pistils from normal flowers as controls. This approach was adopted to compare the differences in internal tissue structure between the control and experimental groups. The samples were fixed in FAA solution for more than 24 h, followed by dehydration, paraffin infiltration, and embedding. Paraffin sections were prepared, and then slides were mounted and dried in an oven at 37 ℃ for subsequent use. The sections were dewaxed, stained with PAS solution in the dark for 25 min, dehydrated, clarified, and mounted with a neutral resin. Examination under an optical microscope revealed that the polysaccharides appeared purple-red.

We collected vegetative propagules from branching flowers for the experimental group, while using pistils from normal flowers as the control group. Using scanning electron microscopy (Hitachi, SU8100, Japan) to observe the upper surface of the vegetative propagules (experimental group) and the surface of the stigma (control group). This approach was adopted to compare the surface morphological characteristics between the control and experimental groups.

Fresh samples were fixed in electron microscope fixative at 4 ℃ for over 24 h. Subsequent dehydration was performed using anhydrous ethanol and isopentyl acetate at various concentrations. The samples were then dried in a critical dryer before gold spraying to enhance the conductivity, followed by examination under scanning electron microscopy.

### Endogenous hormone detection

After grinding with liquid nitrogen, 150 mg of the sample was accurately weighed, and 4 µL of internal standard solution and 150 µL of 80% acetonitrile water (containing 1% formic acid) were added, mixed well, and extracted through immersion at 4 ℃ for 16 h. The sample was sonicated for 20 min in an ice-water bath, and 246 µL of water was added, mixed well, and centrifuged for 15 min (20,000 rcf, 4 ℃). The supernatant was added to a 1.5 mL ep-tube and then centrifuged for 15 min (20,000 rcf, 4 ℃). After which, 150 µL of supernatant was added to an injection vial for LC-MS/MS analysis.

Target compounds were separated and quantified using a Waters Acquity UPLC system coupled to an AB Sciex Triple Quad 5500 + mass spectrometer. Chromatographic separation was carried out on an Agilent Poroshell 120 EC-C18 (100 × 2.1 mm, 2.7 μm) column at 40 ℃. The mobile phase consisted of 0.1% formic acid in water and acetonitrile. The injection volume was 7 µL. The data were acquired under multiple reaction monitoring mode with ESI ion source. The instrumental parameters were optimized for maximal sensitivity with the voltage and source and gas settings as follows: ion spray voltage (IS): 4500 V in positive mode and − 4500 V in negative mode; curtain gas (CUR): 20 psi; collision gas (CAD): 9; temperature (TEM): 400 ℃; ion source gas 1 (GS1): 50 psi; ion source gas 2 (GS2): 50 psi.

### Non-targeted metabolomic analysis

Samples T1, T2, T3, T4, and CK were tested through non-targeted metabolomics by LC Bio Technology Co., Ltd. (https://www.lc-bio.com/). The collected samples were thawed on ice, and metabolites were extracted with 80% methanol buffer. Briefly, 50 mg of sample was extracted with 0.5 ml of precooled 80% methanol. The extraction mixture was then stored in 30 min at -20 ℃. After centrifugation at 20,000 g for 15 min, the supernatants were transferred into new tubes, then vacuum dried. The samples were redissolved with 100 µL 80% methanol and stored at -80 ℃ prior to LC-MS analysis. In addition, pooled QC samples were also prepared by combining 10 µL of each extraction mixture.

Mobile phase conditions: All samples were analyzed using the LC-MS system as detailed below. Firstly, all chromatographic separations were performed using an UltiMate 3000 UPLC System (Thermo Fisher Scientific, Bremen, Germany). An ACQUITY UPLC T3 column (100 mm * 2.1 mm, 1.8 μm, Waters, Milford, USA) was used for the reversed phase separation. The column oven was maintained at 40 ℃. 5 mM ammonium acetate and 5 mM acetic acid and solvent B (acetonitrile). Flow rate was 0.3 ml/min and the mobile phase consisted of solvent A. Gradient elution conditions were set as follows: 0ཞ0.8 min, 2% B; 0.8ཞ2.8 min, 2% to 70% B; 2.8ཞ5.6 min, 70% to 90% B; 5.6 ~ 6.4 min, 90% to 100% B; 6.4 ~ 8.0 min, 100% B; 8.0ཞ8.1 min, 100% to 2% B; 8.1ཞ10 min, 2% B.

A high-resolution tandem mass spectrometer TripleTOF 6600 (SCIEX, Framingham, MA, USA) was used to detect metabolites eluted from the column. The Q-TOF was operated in both positive and negative ion modes. The curtain gas was set 30 PSI, Ion source gas1 was set 60 PSI, Ion source gas2 was set 60 PSI, and an interface heater temperature was 500 ℃. For positive ion mode, the Ionspray voltage floating were set at 5000 V, respectively. For negative ion mode, the Ionspray voltage floating were set at -4500 V, respectively. The mass spectrometry data were acquired in IDA mode. The TOF mass range was from 60 to 1200 Da. The survey scans were acquired in 150 ms and as many as 12 product ion scans were collected if exceeding a threshold of 100 counts per second (counts/s) and with a + 1 charge state. Dynamic exclusion was set for 4 s. During the acquisition, the mass accuracy was calibrated every 20 samples.

VIP scores were derived through multivariate statistical partial least squares discrimination analysis (PLS-DA) analysis, and the final significant differential metabolites were screened based on the simultaneous fulfilment of three conditions: *P* < 0.05 from t-test, fold change ≥ 1.2 or fold change ≤ 1/1.2, and VIP ≥ 1 calculated from PLS-DA analysis. The screened differential metabolites were annotated using the Kyoto Encyclopedia of Genes and Genomes (KEGG) database (http://www.kegg.jp/kegg/compound/), and then the differential metabolites were mapped to the KEGG Pathway database for analysis.

### RNA extraction, library construction, and sequencing

Total RNA from T1, T2, T3, T4, and CK samples was extracted using TRIzol reagent (Thermo Fisher, 15596018). The amount and purity of the total RNA were quality-controlled using a NanoDrop ND-1000 system (NanoDrop, Wilmington, DE, USA), and RNA integrity was examined using a Bioanalyzer 2100 (Agilent, CA, USA). High-quality RNA samples (RIN > 7.0) were used to construct the sequencing library. mRNA was purified using oligo (dT) magnetic beads for cDNA preparation. cDNA was synthesized through reverse transcription following RNA fragmentation, amplified by ligating sequencing junctions, and used to construct 15 transcriptome libraries. Finally, we performed 2 × 150 bp paired-end sequencing (PE150) on an Illumina NovaSeq 6000 system (LC-Bio Technology Co., Ltd., Hangzhou, China), following the manufacturer’s recommended protocol.

Reads containing aptamer contamination, low-quality bases, or undetermined bases were removed using Cutadapt (v.1.9). Sequence quality was verified using FastQC (http://www.bioinformatics.babraham.ac.uk/projects/fastqc/ 0.10.1), including the Q20, Q30, and GC contents of the clean data. All subsequent analyses were based on clean high-quality data.

We attempted reference-based transcriptome sequencing of the *N. colorata* genome [2], but the alignment rate was very low (Supplementary Table 1). Therefore, transcript sequences were obtained through de novo transcription assembly using Trinity (v.2.15). Sequences were spliced, redundancy was eliminated, and expression matrices were generated following quantitative analysis. All assembled single genes were compared to the non-redundant (Nr) protein (http://www.ncbi.nlm.nih.gov/), Gene Ontology (GO) (http://www.geneontology.org), SwissProt (http://www.expasy.ch/sprot/), KEGG (http://www.genome.jp/kegg/), and eggNOG (http://eggnogdb.embl.de/) databases. For identification and analysis of differentially expressed genes (DEGs), we used Salmon (1.9.0) to calculate expression levels of single genes by calculating TPM (transcripts per kilobase per million mapped reads exon model) to assess the length and depth of sequencing. DEGs were identified using the R package edgeR (3.40.2), with log2 > 1 or log2 < − 1 and false discovery rate < 0.05.

### Co-expression network analysis

Co-expression networks were constructed using the WGCNA (v.1.47) package in R. After filtering genes with expression levels < 10, the gene expression values were imported into WGCNA to construct co-expression modules using the automatic network construction function blockwiseModules with default settings. As shown in Supplementary Fig. 1, after power reaches 17, Scale Free Topology Model Fit (R^2^) reaches more than 0.8, and R^2^ remains basically stable. At the same time, the mean connectivity of the network also tends to be stable. Therefore, the soft threshold power is set to 17. TOM Type (topological overlap matrix) was unsigned, mergeCutHeight was 0.25, and minModuleSize was 50. Pearson’s correlation analysis was used to identify modules that were significantly associated with metabolic traits and endogenous hormones (Pearson’s correlation coefficient >|0.65|). Finally, the module network was mapped using Cytoscape 3.7.2. The gene pairs with the top 100 weight values in the module were used for network visualization, and the genes with higher degree values and located in the center of the network are used as hub genes.

### Quantitative real-time PCR analysis

To verify the RNA-Seq data, the expression patterns of 15 related genes were analyzed using real-time fluorescence quantitative PCR (Roche Diagnostic Products (Shanghai) Co., Ltd., Switzerland). *GAPDH* was used as an internal reference gene. The 20 µL reaction volume contained 1 µL of diluted cDNA, 0.4 µL of forward and reverse primers (10 µM), 10 µL of 2 × Universal SYBR qPCR Master Mix (Vazyme, China), and 8.2 µL of ddH_2_O. PCR amplification was performed at 95 °C for 300 s, followed by 40 cycles at 95 °C for 15 s, 60 °C for 15 s, and then 72 °C for 15 s. Three independent biological replicates were established. Sequences of the gene-specific primers are presented in Supplementary Table 2.

## Results

### Microscopic comparison of normal and branching flowers

**Fig. 2:**
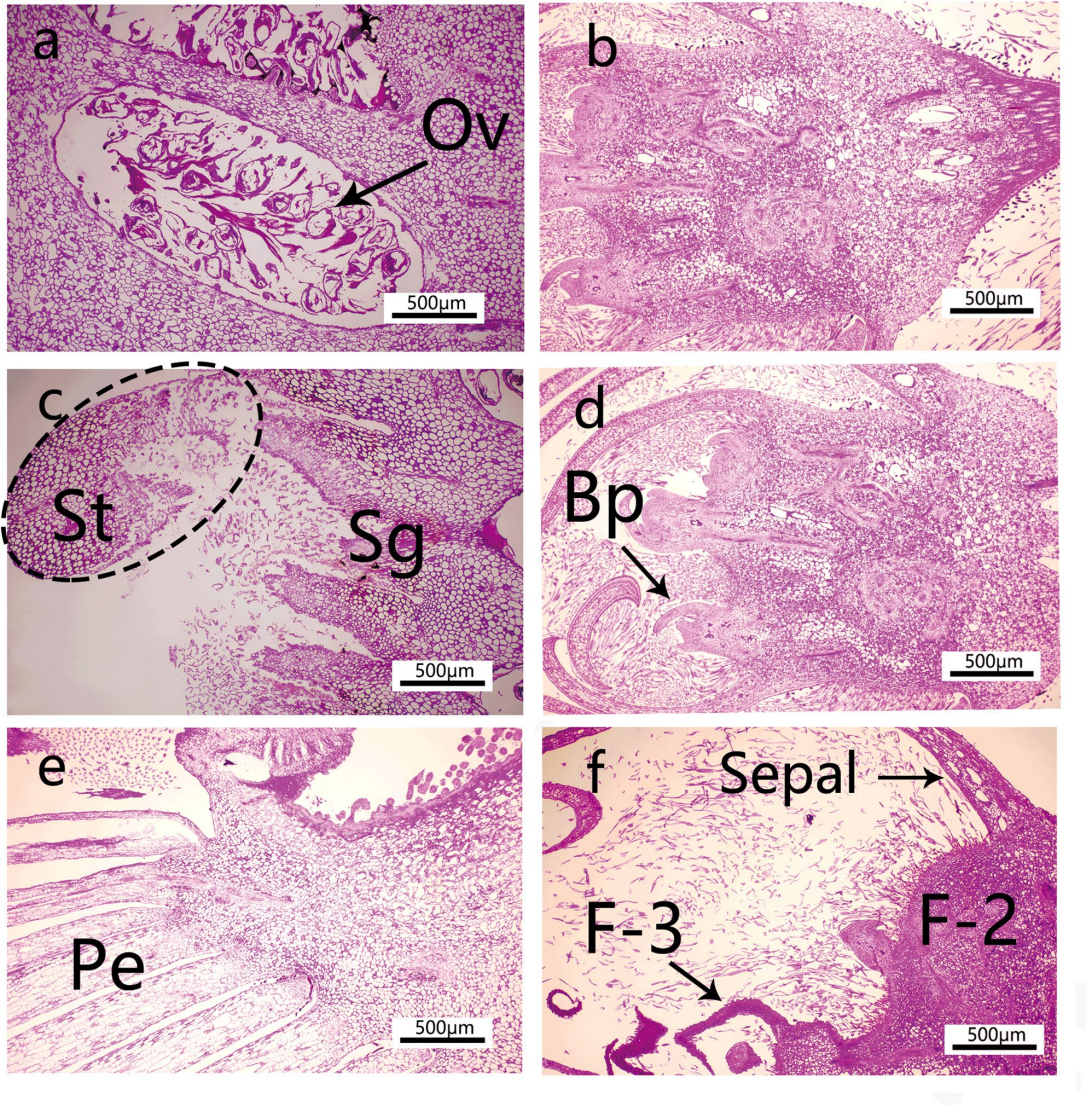
Longitudinal sections of paraffin-embedded slices of normal and second generation of branching flowers. a Normal flowers possess complete pistil tissue, including the ovary (Ov) and ovules within its locules. **b** Vegetative propagule in the second generation of branching flowers. Comparison with Figure **a** In normal flowers, this structure is the pistil, which contains an ovary (Ov); **c** Stigma (Sg) tissue of the pistil and stamens of a normal flower bud, the circle part is stamen (St), and the rest is stigma (Sg) tissue of pistil; **d** New buds sprouting from vegetative propagules. For comparison with Figure **c** In a normal flower, this region bears the stigma (Sg) and stamen (St), Bp: Bud primordium; **e** Pistil of normal flower differentiating into petals (Pe) at its margins. **f **Third-generation branch flowers already differentiating from the second-generation branch flowers. F-2: Second generation of branching flowers; F-3: Third generation of branching flowers. The upper part of a flower is at the left side of the image, and the bottom part of a flower is at the right side of the image

As evident from the specimen collection, the branching flowers exhibited marked differences from the normal flowers, particularly in that the pistil tissue of the branching flowers was entirely replaced by vegetative propagules (Fig. 1). We systematically compared the differences between the pistils of normal flowers and the vegetative propagules of branching flowers using paraffin section analysis. Normal flowers developed pistil tissue above the flower stalk, with the ovary containing several elliptical chambers. Ovoid ovules were attached to ovarian chambers (Fig. 2a). This structural characteristic is the typical morphological basis for sexual reproduction in *N. prolifera*, which ensures the normal development of gametes and fertilization processes, and the loss of this structure in branching flowers directly indicates the loss of sexual reproductive capacity of the flower.

The primary branching flowers are excessively large, such that their overall structure cannot be observed under the microscope; only partial tissues of the vegetative body can be examined, which hampers a clear structural comparison with normal flowers. To visualize the complete architecture of branching flowers and obtain more morphological information, we focused the light microscope on the smaller vegetative propagules within secondary branching flowers (F-2), thereby enabling intact observation of the entire vegetative propagule. Compared with normal flowers, the tissue structure in the ovary region of the branching flowers underwent modification, transforming into a vegetative propagule with more abundant parenchyma tissue (Fig. 2b). The abundant parenchyma tissue is the key structural basis for the vegetative propagules to store nutrients, which provides material support for the subsequent development of the propagules into tubers and the formation of new plants, reflecting the transformation of the flower from a reproductive organ to a vegetative organ. The stigma surface of a normal flower comprised several conical structures. The stigma margin differentiated into stamen tissue (Fig. 2c). Compared with normal flowers, the vegetative propagules of branching flowers continuously formed bud primordia, which subsequently developed into leaves or the next generation of branching flowers (F-2) (Fig. 2d). In the second generation of branching flower buds, the floral primordia of the third generation of branching flowers (F-3) were formed (Fig. 2f). The continuous formation of bud primordia is the core morphological characteristic of the vegetative propagules with asexual reproduction ability, which directly enables the branching flowers to produce new individuals through vegetative propagation and realize the continuous generation of branching flowers (F-2, F-3). This sequential formation of multi-generation floral primordia further confirms the high efficiency of asexual reproduction in *N. prolifera*, which is a unique adaptive strategy for the species to expand its population. The petals of the normal flowers were attached to the epidermal tissue of the pistils (Fig. 2e). Compared with normal flowers, branching flowers were unable to differentiate petals, producing only sepal tissue (Fig. 2f). The loss of petal differentiation and the only development of sepal tissue indicate that the branching flowers have abandoned the morphological traits adapted for sexual reproduction, and the floral organ development program has been completely transformed into a vegetative propagation development program.

**Fig. 3:**
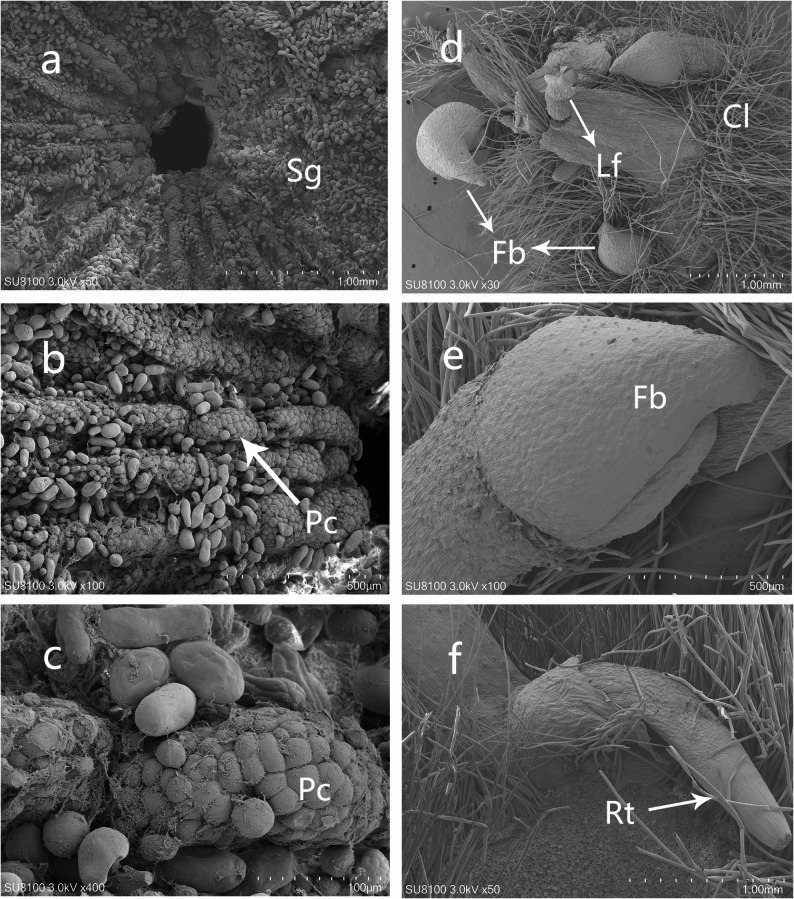
Scanning electron micrographs of normal and branching flowers. **a** Stigma (Sg) of normal flowers, observation at 50x magnification; **b** Papilla cells (Pc) on the stigma, observation at 30x magnification; **c** Papillary cells arranged closely, observation at 400x magnification; **d** Surface of vegetative propagules, Fb: Flower bud; Lf: Leaf Bud; Cl: Cilia; Observation at 30x magnification; **e** Morphology of branching floral bud (Fb), observation at 100x magnification; **f** Vegetative propagules differentiating into root tip tissue, Rt: Root tip; Observation at 50x magnification

The surface regions of the stigma in normal flowers and the upper surface of vegetative propagules in branching flowers were examined using scanning electron microscopy. The stigma of normal flowers had a central aperture with striations distributed across its surface (Fig. 3a). The stigma contained numerous granular papillary cells arranged in close clusters, presumably facilitating pollen capture (Fig. 3b, c). Compared to the stigma of a normal flower, the surface microstructure of vegetative propagules in branching flowers was entirely different, bearing flower buds, leaf buds, and numerous cilia (Fig. 3d). The leaf buds primarily exhibited a curled triangular shape (Fig. 3d) and gradually unfurled over time to form leaves. The flower buds were predominantly present as conical protuberances enclosed by two sepals (Fig. 3e). Vegetative propagules differentiated into root tip tissues (Fig. 3f). Microscopic observations indicate that the reproductive organs of branching flowers are completely transdifferentiated into vegetative propagules, losing sexual reproductive capacity.

### Analysis of endogenous hormones in *N. prolifera* normal and branching flowers

**Fig. 4:**
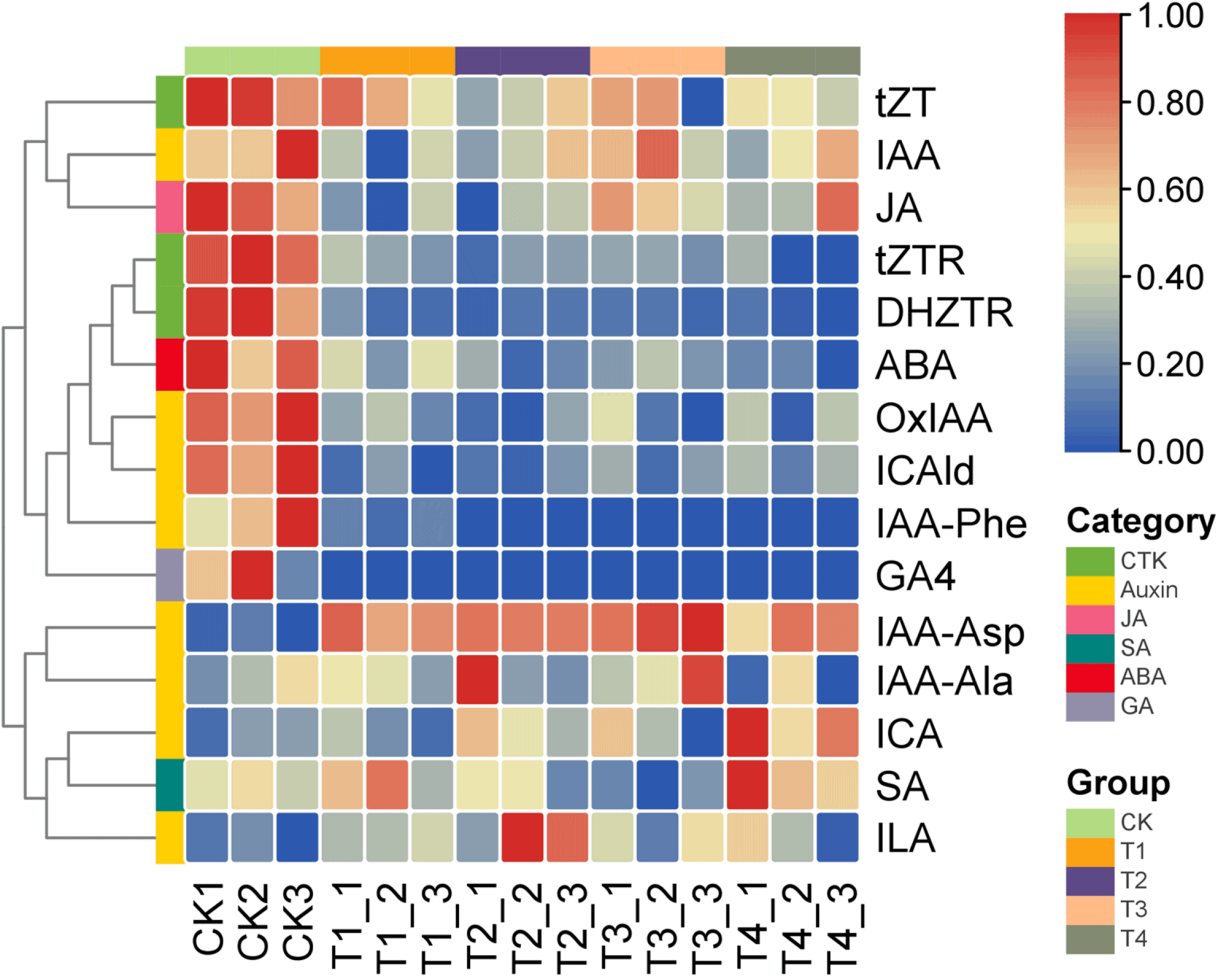
Heatmaps of 15 endogenous hormones in T1, T2, T3, T4, and CK samples. Each sample included three biological replicates. The hormone content values were normalized by the zero to one method, and samples were clustered based on the content of each hormone using the complete linkage method with Euclidean distance. The color gradient represents the relative content of endogenous hormones (red: high content, blue: low content). Abbreviations: tZT: trans-zeatin; tZTR: trans-zeatin riboside; DHZTR: dihydrozeatin riboside; OxIAA: oxindole-3-acetic acid; IAA-Asp: indole-3-acetyl-L-aspartic acid; ICA: indole-3-carboxylic acid; ICAld: indole-3-carboxaldehyde; IAA: indole-3-acetic acid; IAA-Phe: indole-3-acetyl-L-phenylalanine; JA: jasmonic acid; SA: salicylic acid; ILA: indole-3-lactic acid; IAA-Ala: indole-3-acetyl-L-alanine; ABA: abscisic acid; GA_4_: gibberellin acid 4

We detected 15 endogenous hormones in T1, T2, T3, T4, and CK using LC-MS methodology. These included three CTKs, eight auxins, jasmonic acid, salicylic acid, abscisic acid, and gibberellins. Regarding CTKs, the tZTR and DHZTR contents in the CK were significantly higher than those in the treatment groups (*P* < 0.0001, and P-values are nominal) (Supplementary Fig. 2). Concerning auxins, the OxIAA, ICAld, and IAA-Phe contents in the CK were significantly higher than those in the treatment groups, particularly with IAA-Phe content undetectable in T2, T3, and T4. However, IAA-Asp content in the treatment groups was significantly higher than that in the CK (Fig. 4). Different auxins show markedly distinct levels in normal and branching flowers, with OxIAA, ICAld and IAA-Phe higher in normal flowers and IAA-Asp elevated in branching flowers. The ABA content of the CK was significantly higher than that in the treatment groups. GA_4_ was detected in normal flowers, whereas no GA_4_ was detected in branching flowers. Therefore, ABA and GA₄ may be positively associated with the formation of normal flowers in water lilies. The results of endogenous hormone detection revealed significant differences in eight hormone compounds between branching flowers and normal flowers, including 2 cytokinins, 4 auxin-related compounds, ABA, and GA₄.

### Comparison of metabolic profiles of *N. prolifera* normal and branching flowers

A total of 439 metabolites were identified in the vegetative propagules of branching flowers at different developmental stages and in the pistils of normal flowers. Lipids and lipid-like molecules were the most abundant (180), accounting for 41% of the total. The next largest group was phenylpropanoids and polyketides (82), accounting for 18.68% of the total. Organic acids and derivatives followed (43), accounting for 9.8% of the total (Supplementary Fig. 3a). In the PLS-DA of these metabolites, the first principal component was 21.56%, and the second principal component was 8.16% (Supplementary Fig. 3b). The CK samples were clearly distinct from the treatment samples. The T1, T2, T3, and T4 treatment groups tended to spread from the center to the first quadrant, reflecting the differences in metabolites between normal and branching flowers and the evolution of metabolites at different developmental stages of the trophic propagules. The QC samples were concentrated at one point, indicating the high reliability of the results. The replacement test plot showed that the model was not overfitted when the horizontal coordinates were within [0, 1], the R2 regression line was above Q2, and the intercept between the Q2 regression line and the Y-axis was less than 0 (− 0.629), indicating that the test results were reliable (Supplementary Fig. 4).

**Fig. 5:**
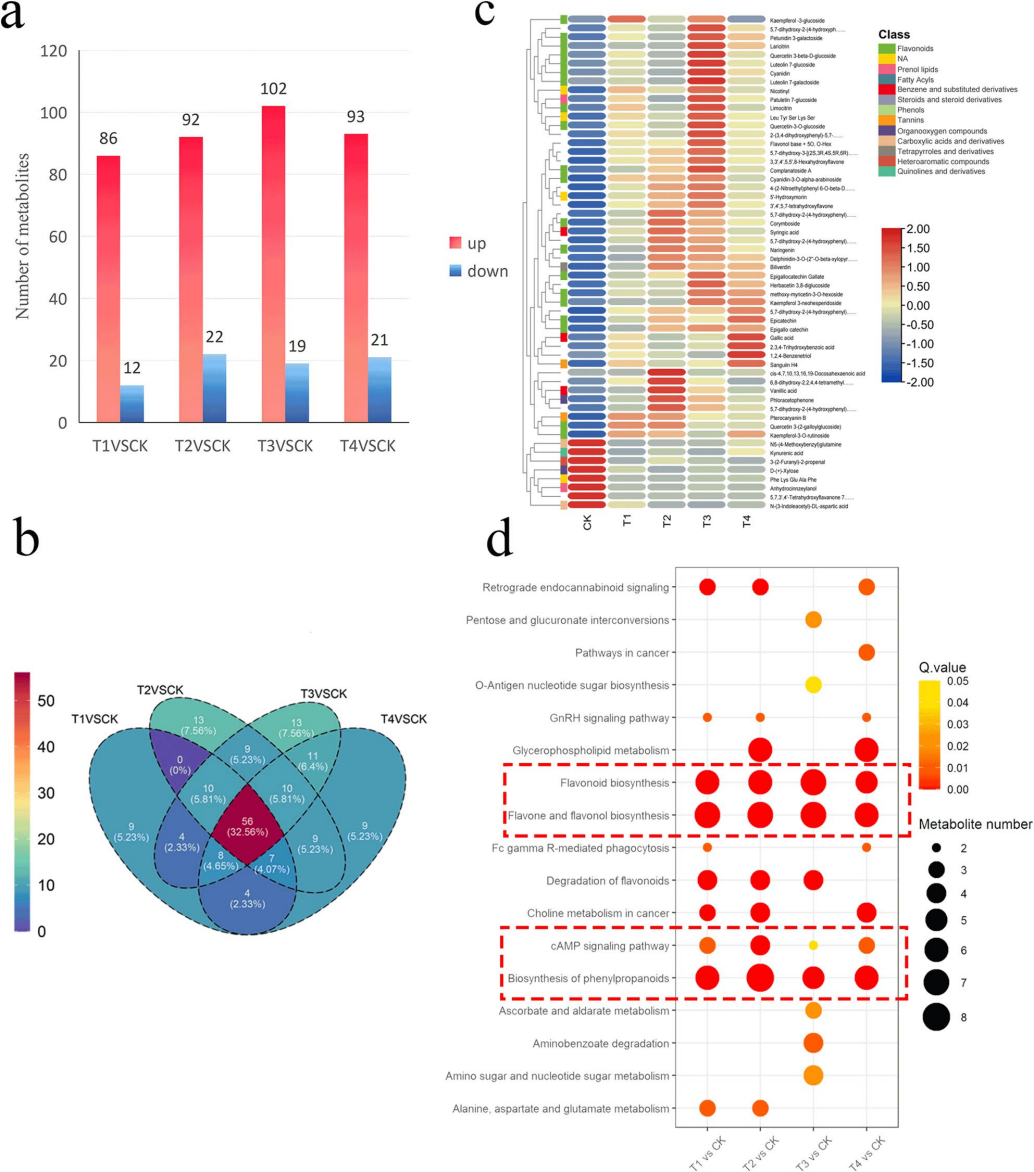
Differentially accumulated metabolites (DAMs) in T1, T2, T3, T4, and CK. **a** Statistical summary of DAMs. **b** Venn diagrams of differentially expressed metabolites of T1 vs. CK, T2 vs. CK, T3 vs. CK, and T4 vs. CK. **c** Heatmap analysis of 56 common metabolites shared between T1 vs. CK, T2 vs. CK, T3 vs. CK, and T4 vs. CK. **d** KEGG enrichment analysis of DAMs in T1 vs. CK, T2 vs. CK, T3 vs. CK, and T4 vs. CK

The differentially accumulated metabolites (DAMs) in T1 vs. CK, T2 vs. CK, T3 vs. CK, and T4 vs. CK were analyzed (Fig. 5). In total, 98 DAMs were identified between the T1 and CK treatments, including 86 up-regulated and 12 down-regulated DAMs. In T2 vs. CK, 92 DAMs were up-regulated and 22 were down-regulated. For T3 vs. CK, 102 DAMs were up-regulated and 19 were down-regulated. For T4 vs. CK, 93 DAMs were up-regulated and 21 were down-regulated. Generally, the expression of most metabolites was up-regulated in vegetative propagules (Fig. 5a). In the Venn analysis, 172 DAMs were identified across all comparison groups, with 56 being common to T1 vs. CK, T2 vs. CK, T3 vs. CK, and T4 vs. CK, accounting for 32.56% of the total (Fig. 5b). Among the 56 metabolites, 31 were flavonoids that were highly expressed in vegetative propagules, particularly in those from T3. Heteroaromatic compounds, organic oxygen compounds, carboxylic acids and derivatives, and quinolines and derivatives were highly expressed in flower pistils (Fig. 5c).

KEGG pathway analysis of the DAMs was conducted to identify significantly enriched metabolic pathways. The differential metabolites of T1 vs. CK were significantly enriched in the flavone and flavonol biosynthesis (ko00944), flavonoid biosynthesis (ko00941), and biosynthesis of phenylpropanoids (ko01061) pathways (Supplementary Fig. 5a). For T2 vs. CK, differential metabolites were significantly enriched in the flavone and flavonol biosynthesis (ko00944), biosynthesis of phenylpropanoids (ko01061), and choline metabolism in cancer (ko05231) pathways (Supplementary Fig. 5b). In T3 vs. CK, differential metabolites were significantly enriched in the flavone and flavonol biosynthesis (Ko00944), flavonoid biosynthesis (ko00941), and degradation of flavonoids (ko00946) pathways (Supplementary Fig. 5c). For T4 vs. CK, differential metabolites were significantly enriched in choline metabolism in cancer (ko05231), flavone and flavonol biosynthesis (Ko00944), and glycerophospholipid metabolism (ko00564) pathways (Supplementary Fig. 5d).

The results showed that most DAMs were enriched in four metabolic pathways: flavonoid biosynthesis, flavone and flavonol biosynthesis, cAMP signaling pathway, and phenylpropanoid biosynthesis, most of which were up-regulated in all four comparisons (Fig. 5d). The pistil of normal flowers transforms into a vegetative propagule, forming a unique branching flower. From the KEGG enrichment analysis of the metabolome, it appears that flavonoids were more abundant in the vegetative propagules.

### Comparing transcriptomes of *N. prolifera* between normal and branching flowers

To investigate the molecular mechanisms underlying branching flower formation, we sequenced the transcriptome of vegetative propagules from four developmental stages of branching flowers, as well as samples from the ovary site of normal flowers. We constructed 15 cDNA libraries and generated 5.15–6.34 G of data per sample. The GC content of each sample ranged from 46.08% to 51.06%, with Q20 base percentages exceeding 97.73% and Q30 base percentages exceeding 93.07% (Supplementary Table 3).

All samples were assembled using Trinity, and 94,503 genes were obtained. We annotated a total of 1611 transcription factors, and their expression profiles are shown in Supplementary Fig. 6. The percentage of GC content was 42.63%, the shortest gene length was 177, the median gene length was 1296, the longest gene length was 16,822, the total number of bases assembled was 87,690,151, and the N50 length was 2035 (Supplementary Table 4). Stringent thresholds of absolute log2 FC ≥ 1 and false discovery rate < 0.05 were used to screen DEGs. GO enrichment and KEGG pathway analysis were applied to assay these DEGs to understand their biological function. Twelve genes were randomly selected to test the reliability and consistency of transcriptome results. The expression levels of these genes were assessed using qRT-PCR. The expression patterns obtained through qRT-PCR and RNA-seq analyses were similar, with Pearson correlation coefficients > 0.7 for both (Supplementary Fig. 7).

**Fig. 6:**
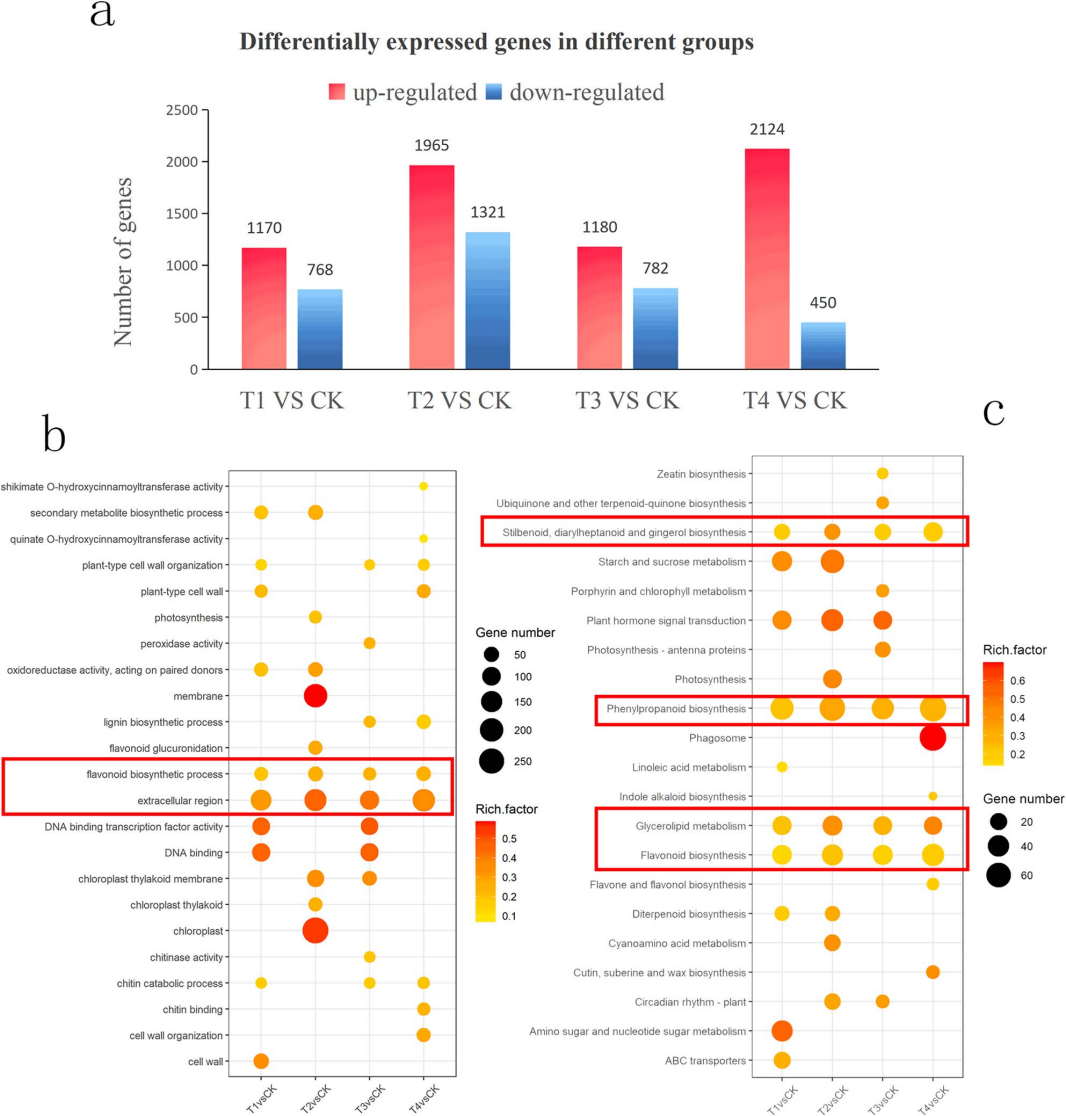
Analysis of differentially expressed genes (DEGs) in T1, T2, T3, T4, and CK. **a** Number of up-regulated and down-regulated genes in four comparison groups. **b** Gene Ontology (GO) terms enriched in different comparisons displaying the top 10 GO terms with *P* < 0.01. The GO terms enclosed by red squares are the common enriched items across the four comparison groups. **c** Kyoto Encyclopedia of Genes and Genomes (KEGG) pathways enriched in different comparisons displaying the top 10 KEGG pathways with *p* < 0.01. The KEGG pathways enclosed by red squares are the common enriched items across the four comparison groups

To investigate the candidate genes contributing to normal and branching flowers in *N. prolifera*, we investigated different developmental stages of branching flowers, with T1, T2, T3, and T4, as experimental groups, and normal flowers as the control group CK, and established four comparison groups: T1 vs. CK, T2 vs. CK, T3 vs. CK, and T4 vs. CK. Many DEGs were identified among the four comparison groups, with 1938 in T1 vs. CK, including 1170 up-regulated and 768 down-regulated genes. A total of 3,286 genes were detected in T2 vs. CK, of which 1,965 were up-regulated and 1,321 were down-regulated. In the T3 vs. CK test, 1962 genes were detected, of which 1180 were up-regulated and 782 were down-regulated. A total of 2574 genes were detected in the T4 vs. CK comparison group, including 2124 up-regulated and 450 down-regulated genes (Fig. 6a) (Supplementary Table 5). The top 50 induced and top 50 suppressed genes from the four comparison groups are shown in Supplementary Fig. 8. In all four comparison groups, the number of up-regulated DEGs was significantly higher than that of down-regulated ones. These results suggested that the up-regulated DEGs play crucial roles in supporting the development of vegetative propagules.

To predict the function of the DEGs in branching flowers, GO enrichment analysis was performed on the DEGs from the four comparison groups. The DEGs in all groups were enriched in flavonoid biosynthetic process and extracellular region terms (Fig. 6b). In the term flavonoid biological process, the number of DEGs in the T1 vs. CK group was 36 (rich factor = 0.19), 48 DEGs were enriched in T2 vs. CK (rich factor = 0.25), 31 DEGs were enriched in T3 vs. CK (rich factor = 0.23), and 43 DEGs were enriched in T4 vs. CK (rich factor = 0.23). In the extracellular region term, the number of DEGs in T1 vs. CK, T2 vs. CK, T3 vs. CK, and T4 vs. CK was 143, 170, 109, and 172, respectively. The enrichment factors were 0.31, 0.45, 0.42, and 0.38, respectively. These results indicated that the formation of branching flowers is closely related to flavonoid synthesis and genes with extracellular domain functions.

KEGG enrichment analysis identified the metabolic pathways and functions of the DEGs. Based on the KEGG enrichment analysis of the four comparison groups, all were significantly enriched in four metabolic pathways: flavonoid biosynthesis (map00941), phenylpropanoid biosynthesis (map00940), glycerolipid metabolism (map00561), and stilbenoid, diarylheptanoid, and gingerol biosynthesis (map00945). In addition, the plant hormone signal transduction pathway was enriched in T1 vs. CK, T2 vs. CK, and T3 vs. CK, and the enrichment factors were all > 0.44 (Fig. 6c, Supplementary Table 6). The expression heatmap of genes involved in the plant hormone signal transduction pathway is shown in Supplementary Fig. 9a. Most hormone signaling-related genes exhibited low expression levels during branching flower development. We hypothesize that the branching flower formation stage may be related to endogenous hormone transmission.

Transcriptome analysis of *N. prolifera* between normal and branching flowers showed that most differentially expressed genes in branching flowers were up-regulated, and the formation of branching flowers was closely related to flavonoid synthesis, glycerolipid metabolism, stilbenoid, diarylheptanoid, and gingerol biosynthesis and plant hormone signal transduction.

### Analysis of DEG expression related to flavonoid biosynthesis

**Fig. 7:**
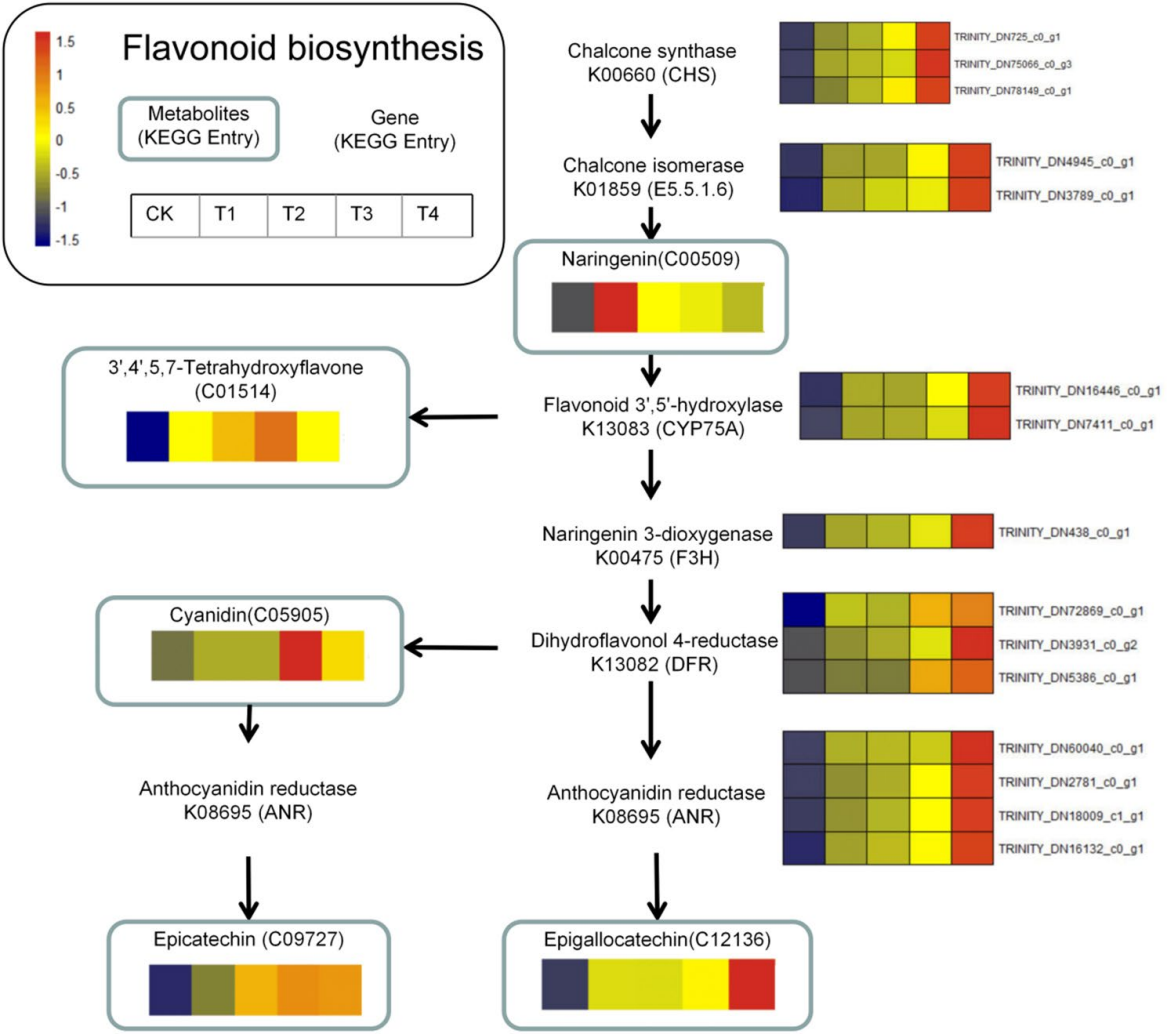
Heatmap of differentially expressed genes and differentially expressed metabolites in flavonoid biosynthesis. The pathway was built based on the Kyoto Encyclopedia of Genes and Genomes (KEGG) pathways

KEGG enrichment analysis of the transcriptome and metabolome data showed that DEGs and metabolites were significantly enriched in the flavonoid biosynthesis pathway. The expression patterns of genes related to the flavonoid biosynthesis pathway are shown in Supplementary Fig. 9b. Most genes involved in flavonoid biosynthesis were highly expressed during the late stages of branching flower development. In particular, naringenin, cyanidin, epigallocatechin, epicatechin, and 3’,4’,5,7-tetrahydroxyflavone were the common DAMs of the four comparison groups, and accumulated more in the vegetative propagule of branching flowers. To further clarify the mechanism underlying flavonoid formation in vegetative propagules, we compared and analyzed five metabolites (naringenin, cyanidin, epigallocatechin, epicatechin, and 3’,4’,5,7-tetrahydroxyflavone) involved in flavonoid biosynthesis with their related DEGs (Fig. 7). The results showed that flavonoid biosynthesis genes and metabolites are co-upregulated in vegetative propagules.

### Identification of WGCNA modules associated with target traits

**Fig. 8:**
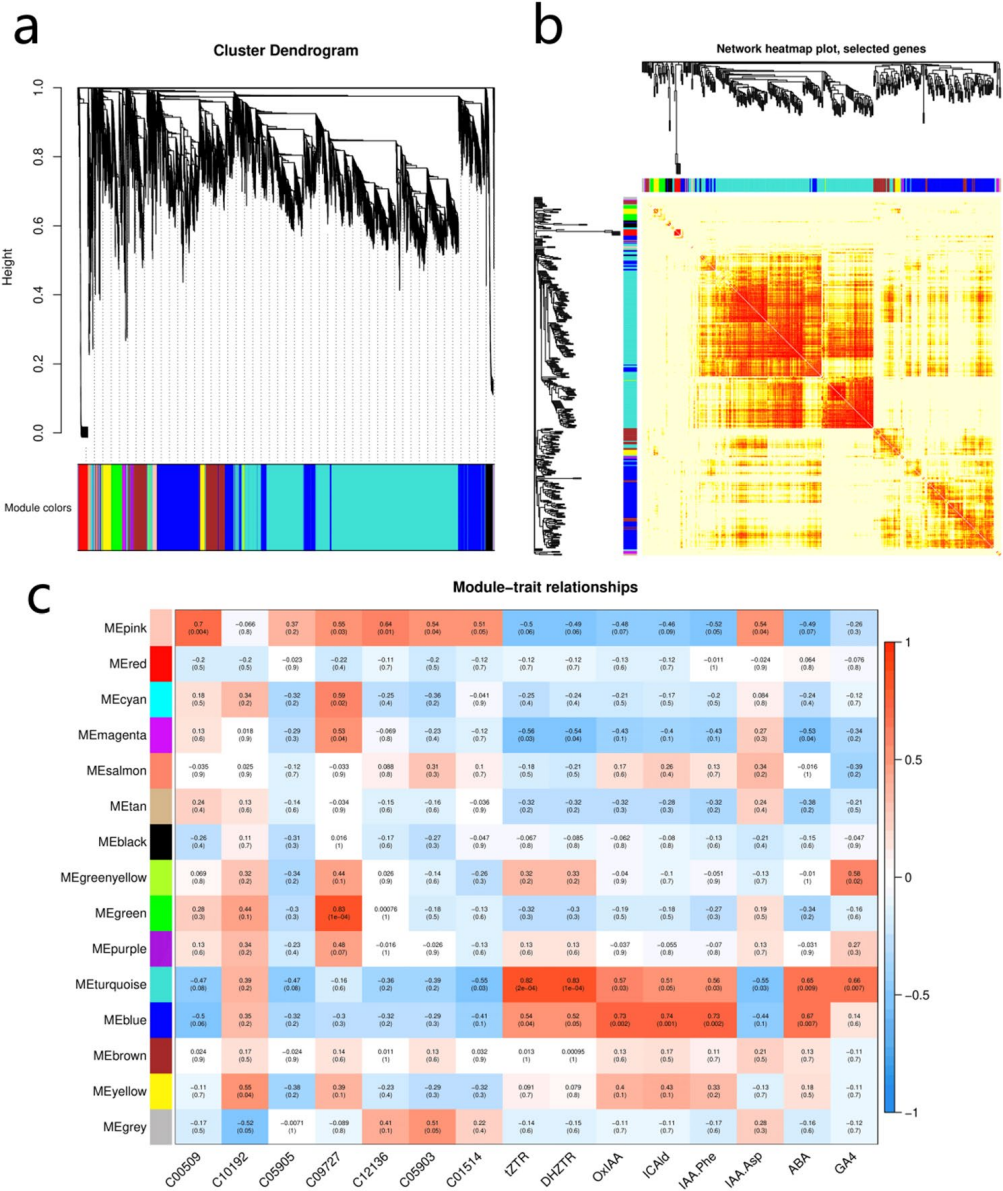
Weighted gene co-expression network analysis of 12,964 genes in normal and branching flowers of *N. prolifera.* **a** Hierarchical clustering tree showing co-expression gene modules. Each leaf of the tree corresponds to one gene, and 15 modules are labeled with different colors. **b** Heatmap of module gene clustering depicting the topological overlap matrix among 1,000 randomly selected genes. Deeper red represents stronger connectivity between the two genes corresponding to the row and column. **c** Association analysis of gene co-expression network modules with flavonoid biosynthesis metabolites and endogenous hormones. The numbers within the heatmap correspond to correlations and p-values (in parentheses). Red represents a positive correlation, and blue represents a negative correlation. C00509: naringenin; C10192: tricetin; C05905: cyanidin; C09727: epicatechin; C12136: epigallocatechin; C05903: kaempferol; C01514: 3’,4’,5,7-tetrahydroxyflavone; tZTR, trans-zeatin riboside; DHZTR: dihydrozeatin riboside; OxIAA: oxindole-3-acetic acid; ICAld: indole-3-carboxaldehyde; IAA.Phe: indole-3-acetyl-L-phenylalanine; IAA.Asp: indole-3-acetyl-L-aspartic acid; ABA: abscisic acid; GA_4_: gibberellin acid 4

Metabolomics and targeted endogenous hormone detection revealed that flavonoid metabolites, such as naringenin, cyanidin, epigallocatechin, and epicatechin, and endogenous hormones, such as tZTR, DHZTR, and OxIAA, were differentially expressed in normal and branching flowers. This process affects the formation and development of branching flowers. To study the co-expression regulatory network of these metabolite-related genes, we filtered the low-expression genes and conducted WGCNA based on the expression of 12,964 filtered genes. Fifteen modules were identified, and each module was marked with a different color (Fig. 8a, b).

To identify which modules were closely associated with the formation of branching flowers, we conducted a correlation analysis between the gene co-expression modules and flavonoid DAMs as well as endogenous hormones identified in prior research (Fig. 8c). The correlation analysis between the gene modules and metabolic traits revealed that the MEgreen module was highly correlated with epicatechin (KEGG ID: C09727), with a correlation coefficient of 0.83. The MEblue module showed a high correlation with the auxins OxIAA, ICAld, and IAA. Phe with correlation coefficients of 0.73, 0.74, and 0.73, respectively. The MEturquoise module exhibited high correlation with CTKs tZTR and DHZTR, with correlation coefficients of 0.82 and 0.83, respectively. Concurrently, MEturquoise was highly correlated with the ABA and GA_4_ (Pearson’s correlation coefficient > 0.65).

**Fig. 9:**
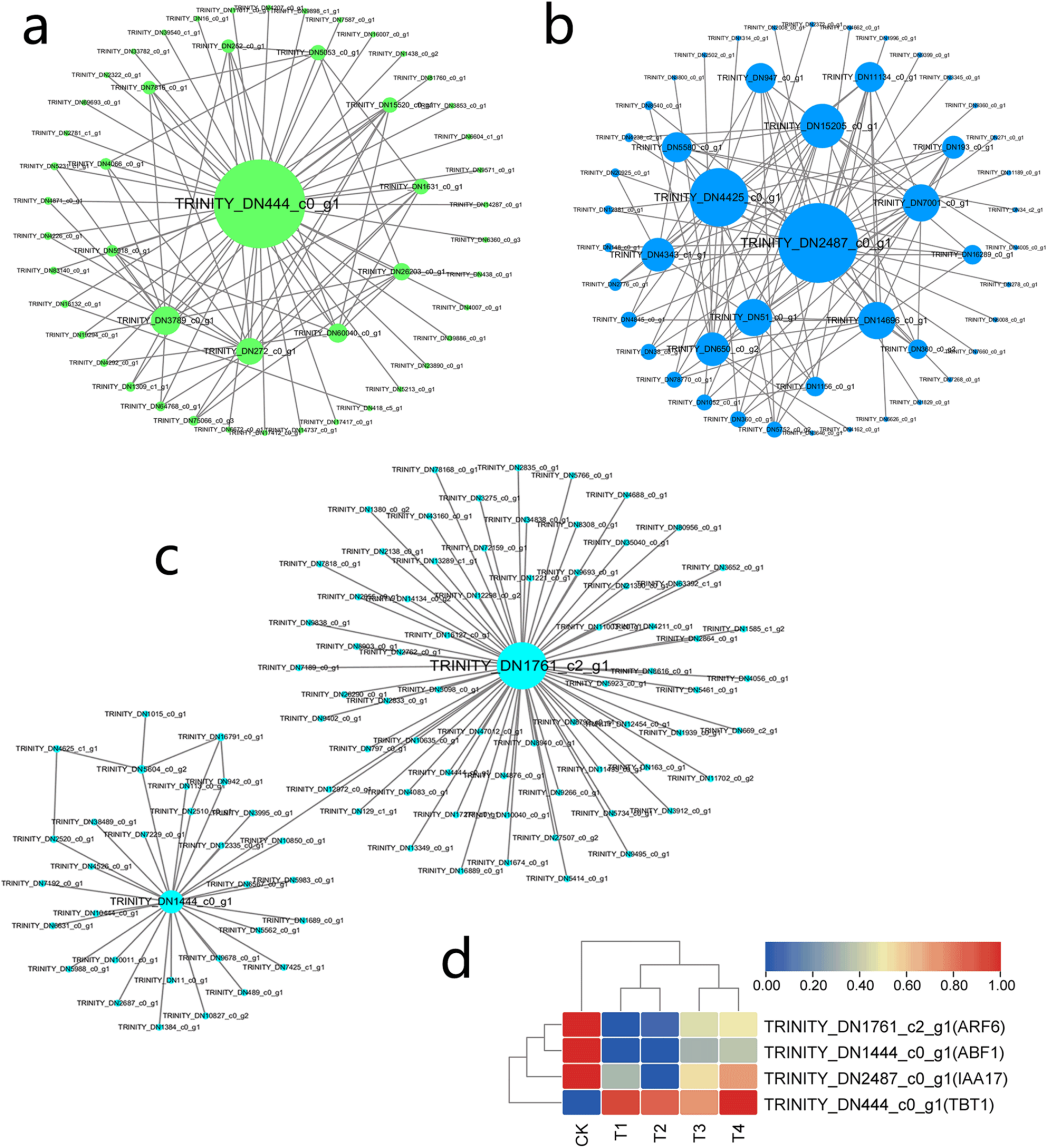
Identification and expression profiling of module hub genes. Gene co-expression network in the green (**a**), blue (**b**), and turquoise (**c**) modules with edge weight of top 100. The size of the node indicates the degree of genes. The genes with higher degree values in each module were considered to be the hub genes. **d** Heatmap of expression for hub genes in each module. Rows were log-scaled and subjected to normalization via the Normalized method

The MEgreen module was highly correlated with epicatechin accumulation, and these module genes were significantly enriched in the flavonoid biosynthesis pathway (Supplementary Fig. 10a). We constructed a co-expression network of this module with genes in the flavonoid biosynthesis pathway and screened hub genes, trinity_dn444_c0_g1, by calculating the degree value between genes (Fig. 9a). This gene was named *TBT1* and annotated as tryptamine hydroxycinnamoyltransferase 2-like in the NR database, tryptamine benzoyltransferase in the Swissport database, and shikimate o-hydroxycinnamoyltransferase in the KOD database. *TBT1* is not a core enzyme for flavonoid biosynthesis. WGCNA clustered most known core flavonoid structural genes into the MEgreen (Supplementary Table 7) *TBT1* showed the highest degree value in this co-expression network. This suggests that *TBT1* may indirectly affect these known structural genes, thereby functioning in flavonoid accumulation.

The MEblue module showed a high correlation with auxin compounds, such as OxIAA, ICAld, and IAA.Phe. This module was significantly enriched in plant hormone signal transduction pathways (Supplementary Fig. 10b). The co-expression network for the MEblue module was constructed using genes from the plant hormone signalling pathway. The hub gene TRINITY_DN2487_c0_g1 was also identified in this network (Fig. 9b). This gene was named *IAA17* and annotated as an auxin-responsive protein in the Nr database and auxin-responsive protein *IAA17* in the SwissProt and KEGG databases.

The MEturquoise module exhibited a high correlation with CTKs such as tZTR and DHZTR. Concurrently, it exhibited strong correlations with ABA and GA_4_. These module genes were significantly enriched in the plant hormone signalling pathway (Supplementary Fig. 10c). Two hub genes, TRINITY_DN1761_c2_g1 (*ARF6*) and TRINITY_DN1444_c0_g1 (*ABF1*), were identified by constructing a co-expression network using genes from this pathway (Fig. 9c). *ARF6*, a member of the auxin response factor (*ARF*) family, emerged as the hub gene with the highest connectivity in the co-expression network. It is annotated as auxin response factor 6 in the Nr database, auxin response factor 6 in the Swiss-Prot database, and auxin response factor in the KOD database. *ABF1* belongs to *bZIP* family and is annotated as an uncharacterized protein LOC116256070 in the NR database, ABSCISIC ACID-INSENSITIVE 5-like protein 4 in the Swiss-Prot database, and ABA-responsive element binding factor in the KEGG database. *ABF1* was identified as the second most highly connected hub gene in this regulatory network, and may play a key regulatory role in abscisic acid accumulation [21].

Through WGCNA, three core modules (MEgreen, MEblue, MEturquoise) were highly correlated with the regulation of hormone or flavonoid metabolism, and genes including *IAA17*,* ARF6*,* TBT1*, etc., were identified as hub genes of these three modules (Fig. 9d).

## Discussion

*N. prolifera* was first discovered in South America by Wiersema in 1984, and occurs in Argentina, Paraguay, Ecuador, and Costa Rica. It derives its name from the profusion of the asexual propagules produced by its flowers. Wiersema described these structures as tuber-bearing “flowers” [4]. In 2006, Valentin Grob described the formation of tuber-bearing ‘flowers’ at the microscopic level (this report described this flower as a branching flower). Grob proposed that *N. prolifera* propagates and sprouts new plants by repeatedly altering the characteristics of the branching flower meristem, continuously transforming it from a floral meristem (FM) to a shoot apical meristem (SAM). This study delves deeper into the microscopic realm [8]. Based on previous research, we conducted a comprehensive analysis of the transcriptome and metabolome of branching flowers at different developmental stages of *N. prolifera* and identified candidate genes associated with branching flower formation.

In the metabolomic and transcriptomic enrichment analyses of this study, we unexpectedly found that flavonoids including epicatechin, naringenin, and 3’,4’,5,7-tetrahydroxyflavone were significantly highly accumulated in branching flowers relative to normal flowers. The expression levels of structural genes encoding key enzymes in the flavonoid biosynthesis pathway (CHS, CHI, F3H, DFR, and ANR) were progressively up-regulated during the development of vegetative propagules, which was consistent with the accumulation trend of flavonoid metabolites (Fig. 7). Combined with the ecological adaptive characteristics of *N. prolifera*, whose vegetative propagules ultimately develop into tubers that give rise to new plants, our findings are consistent with the well-documented general conclusion that flavonoids are abundantly accumulated in plant storage tubers. For example, sweet potato tubers accumulate high levels of flavonoids during their growth and development [22], and the rhizome of lotus (*Nelumbo nucifera Gaertn.*), a major nutrient storage organ, also accumulates substantial amounts of flavonoids during its enlargement [23]. In the present study, genes associated with flavonoid biosynthesis were not only highly expressed in the branching flower, but more intriguingly, their expression levels increased progressively as the branching flower developed (Fig. 9). Using WGCNA, we found that *TBT1* may be a hub regulator of epicatechin accumulation. As branching flowers ultimately detach from the parent plant to form new tubers, we hypothesized that this high accumulation of flavonoids prepares the branching flower for the transition to tuber function.

Plants that fail to complete sexual reproduction under adverse conditions often evolve vegetative propagation strategies [24], which also implies that the vegetative propagules of *N. prolifera* are typically formed under various stressful environments. Flavonoids exhibit strong antioxidant properties and free radical-scavenging activity, serving as key compounds for plant tissues to resist oxidative stress induced by low temperature, salt stress and aquatic hypoxia [25, 26]. Elevated flavonoid levels may afford a protective role in the normal development of vegetative propagules. and act as phytoalexins for plants against biotic stress, inhibiting the infection of pathogenic fungi and bacteria [27, 28]. The pedicels and sepals of branching flowers undergo natural senescence and decay during development, a tissue degradation process that is highly susceptible to microbial infestation. Flavonoid accumulation protects the vegetative propagules from excessive microbial invasion, thereby ensuring their successful development into tubers of new plants.

Beyond stress defense, flavonoids also act as important endogenous signaling molecules in plants that modulate plant hormone signaling pathways. Flavonoids possess auxin transport-inhibiting activity; they can bind to the auxin efflux carriers *PIN* proteins to suppress the polar transport of auxin, thereby altering local auxin gradients and regulating the fate of meristematic cells [29, 30]. Bhakta et al. [31] demonstrated that the *MusaATAF2-like* protein in banana modulates key components of the cytokinin signaling pathway, elicits enhanced plant sensitivity to cytokinins, and promotes flavonoid accumulation, which in turn indirectly represses auxin signaling, thereby regulating shoot apex development and in vitro propagation in banana. These aforementioned studies indicate that flavonoids are not only involved in plant stress defense but also act as downstream targets of transcription factors, participating in the regulation of vegetative growth by modulating plant hormone signaling pathways. In the present study, an integrated analysis of endogenous hormone profiles (Fig. 4) and the flavonoid biosynthetic pathway (Fig. 7) revealed that the levels of most auxins were lower in branching flowers, whereas flavonoid accumulation was consistently elevated in these structures. Collectively, these findings suggest that flavonoids may exert an inhibitory effect on auxins and thus synergistically modulate the development of vegetative propagules.

In addition, the KEGG enrichment analysis of the transcriptome in this study identified the glycerolipid metabolism and stilbenoid, diarylheptanoid and gingerol biosynthesis pathways. Among these, lipids are recognized as essential components of plant cells that provide structural integrity, serve as reservoirs for metabolic energy, and also play an active role in plant defense responses [32, 33]. The stilbenoid, diarylheptanoid and gingerol biosynthesis pathways represent major biosynthetic routes for plant defensive secondary metabolites and are closely associated with the capacity to resist microbial infection [34]. These metabolic pathways are associated with developing vegetative propagules, potentially providing structural support for the rapid cellular proliferation and differentiation of vegetative propagules, supplying material reserves for their subsequent tuberization, and further enhancing the ability of vegetative propagules to resist biotic stress. It should be noted that the aforementioned functional inferences regarding flavonoids and their associated metabolic pathways are solely based on correlative evidence of their high accumulation in branching flowers and the well-characterized functions of flavonoids and these pathways in plants, and direct experimental evidence for such inferences is currently lacking.

Endogenous plant hormones are core regulators of floral bud differentiation and vegetative propagation. Among these, the interaction between auxins and cytokinins is pivotal for maintaining floral meristem (FM) activity and organ development [35]. For example, overexpressing *MusaSNAC1* in banana increases sensitivity to the synthetic auxin 6-benzylaminopurine (6-BA/BAP) in transgenic banana lines and significantly enhances the proliferation efficiency of in vitro banana shoot apices by regulating the expression of key genes in the auxin and cytokinin signalling pathways [36]. Furthermore, signal crosstalk between auxins and cytokinins is directly involved in regional gynoecium specification and ovule formation, which are fundamental to the development of sexual reproductive structures in normal flowers [37]. Cytokinins primarily promote cell division and proliferation in meristematic tissues and maintain the activity of stem cell populations in the shoot apical meristem (SAM) and FM [38–40]. Auxins, on the other hand, govern the determination of the fate of organ primordia and regulate the spatial patterning of floral organs by establishing local concentration gradients [36, 41]. Studies have confirmed that an imbalance between auxins and cytokinins can drive cell proliferation in the floral meristem, ultimately leading to the loss of its meristematic identity [42]. Additionally, the combined action of auxins, gibberellins and cytokinins modulates the size and developmental coordination of the floral meristem, influencing the progress and efficiency of floral bud differentiation [43, 44]. Combined with the results of hormone detection in this study, CTK, GA_4_ and most auxin content were significantly lower in branching flowers at the bud stage (T1 sample) than in normal flowers. We therefore hypothesise that floral bud differentiation and the regionalised development of pistils and ovules in normal *N. prolifera* flowers require higher levels of auxins, CTK and GA_4_. Lower levels of CTK, GA₄ and most auxins may be insufficient to support normal reproductive organ development, which may be associated with the formation of branching flowers.

Using WGCNA, we identified two gene modules that showed strong correlations with the hormone metabolites tZTR, DHZTR, OxIAA, ICAld, and IAA. Phe. Further analysis of the network connectivity within these modules revealed *IAA17*, *ABF1*, and *ARF6* as potential hub genes that regulate the homeostasis of CTKs and auxins.

*IAA17* has been functionally annotated as an auxin-responsive protein. Auxin-dependent transcriptional regulation, which mediates plant growth and development, operates through specific interactions between *ARF* and auxin-responsive proteins. *SlIAA17* interacts with multiple *ARFs*, including *SlARF4*, *SlARF5*,* SlARF6*,* SlARF7*, and *SlARF8* [45]. Furthermore, *SlIAA17* plays a regulatory role in determining fruit size in tomato [46]. In *A. thaliana*, *VvIAA9* overexpression enhances vegetative growth and accelerates floral transition [47]. *ARF* is a plant-specific protein composed of several distinct domains, including an N-terminal DNA-binding domain, a non-conserved middle region, and a C-terminal dimerization domain that facilitates protein–protein interactions [48]. *ARF* is a key regulator of the auxin signaling pathway and plays essential roles in floral development [49]. For instance, in *A. thaliana*, *AtARF3/4* is functionally associated with floral organ morphogenesis and contributes to the regulation of floral development and organ formation [50]. Zhang et al. [51] demonstrated that in *Arabidopsis*, *ARF3* translocates to the central zone of the meristem, where it non-cell-autonomously suppresses CTK level and represses *WUSCHEL* expression, thereby modulating meristem activity. Thus, *ARF3* serves as a molecular link that integrates auxin and CTK signaling in the SAM, coordinating the balance between meristem maintenance and organ initiation. In the present study, the contents of cytokinin (CTK) and most auxins were low in branching flowers, and the transcript levels of *ARF6* and *IAA17* were also reduced, exhibiting a coordinated variation trend. We therefore hypothesize that the down-regulated expression of *ARF6* and *IAA17* may be associated with reduced levels of CTK and major auxins, which may contribute to the disruption of normal floral organ development and the formation of branching flowers. Regarding cytokinin regulation, these observations differ from previous reports in Arabidopsis, where *ARF3* functions to inhibit CTK level. *Nymphaea*, as a basal angiosperm [2], may harbor divergent functions for these genes, which are not equivalent to their roles characterized in model eudicots.

Integrated multi-omics and WGCNA analyses are mainly used to reveal correlations between traits and gene expression. Based on correlation evidence, we identified *IAA17*,* ABF1*,* ARF6* and other genes as potential candidate regulators, which may function in branching flower formation and development by mediating cytokinin, auxin and flavonoid biosynthesis. The causal relationships proposed still need to be further validated by functional experiments targeting *IAA17*,* ABF1*,* ARF6* and related pathways of endogenous hormones and flavonoids in future research.

The results and discussion of this study are all based on a comparative analysis of two heterogeneous organs. The pistils of normal flowers and the vegetative propagules of branching flowers, with the research focus on the transcriptomic and metabolomic differences between these two tissues as well as the dynamic expression changes during the developmental process of branching flowers. This study has certain limitations in elucidating the formation mechanism of branching flowers: although we selected the early-stage sample (T1) at the bud germination stage from tubers for analysis, the vegetative propagules within the flower buds had already initially formed at this time. This phenomenon indicates that the differentiation fate of *N. prolifera* into normal flowers or branching flowers is actually determined at the stage of floral primordium differentiation of maternal plants. Therefore, to more precisely decipher the molecular regulatory mechanism underlying the transition from normal flowers to branching flowers in *N. prolifera*, the sample selection in subsequent studies needs to be further focused on the formation stage of floral primordia in plant tubers, so as to trace the initial molecular regulatory events of branching flower occurrence.

## Conclusions

We employed an integrated approach combining multi-omics and WGCNA to investigate a distinctive reproductive phenomenon in aquatic plants—the formation and development of branching flowers. We systematically compared branching flowers and normal flowers in terms of their microscopic morphology, endogenous hormone profiles, metabolic characteristics and molecular expression patterns. Our results confirmed that branching flowers completely lose pistil tissues and forgo sexual reproductive capacity. Instead, they develop vegetative propagules that generate new individuals via asexual reproduction, enabling the continuous formation of successive generations of branching flowers and thus verifying the high efficiency of asexual reproduction in *Nymphaea prolifera*. A total of 15 endogenous hormones were detected, among which CTKs, ABA, GA₄ and most auxins were significantly reduced in branching flowers, indicating their potential involvement in regulating the formation of branching flowers. In addition, KEGG annotation analysis of transcriptomic and metabolomic data revealed a significant enrichment of the flavonoid biosynthesis pathway in both datasets, suggesting that this pathway may play a role in the formation and development of branching flowers. By constructing a gene co-expression network, we identified three key gene modules that are closely correlated with endogenous hormone metabolism and flavonoid biosynthesis, with *IAA17*, *ARF6*, *ABF1* and *TBT1* identified as the hub genes of these modules. These findings provide a novel perspective for understanding the formation and development of branching flowers in *N. prolifera*.

## Supplementary Information

Supplementary Material 1.

Supplementary Material 2.

Supplementary Material 3.

Supplementary Material 4.

Supplementary Material 5.

Supplementary Material 6.

## Data Availability

All relevant supporting data sets are included in the article and its supplemental files. The raw RNA-seq data have been submitted to the SRA database under accession number PRJNA1347974, and they can also be freely available at: https://www.ncbi.nlm.nih.gov/bioproject/PRJNA1347974.All untargeted metabolomic data reported in this paper have been deposited in the OMIX, China National Center for Bioinformation / Beijing Institute of Genomics, Chinese Academy of Sciences (https://ngdc.cncb.ac.cn/omix:accession: accession no.OMIX015639).

## Abbreviations

WGCNA: Weighted gene co-expression network analysis
LC-MS/MS: Liquid chromatography-tandem mass spectrometry
DEGs: Differentially expressed genes
Nr: Non-redundant protein database
GO: Gene Ontology
KEGG: Kyoto Encyclopedia of Genes and Genomes
TPM: Transcripts per kilobase per million mapped reads exon model
FDR: False discovery rate
tZT: trans-zeatin
tZTR: trans-zeatin riboside
DHZTR: Dihydrozeatin riboside
OxIAA: Oxindole-3-acetic acid
IAA-Asp: Indole-3-acetyl-l-aspartic acid
ICA: Indole-3-carboxylic acid
ICAld: Indole-3-carboxaldehyde
IAA: Indole-3-acetic acid
IAA-Phe: Indole-3-acetyl-L-phenylalanine
JA: Jasmonic acid
SA: Salicylic acid
ILA: Indole-3-lactic acid
IAA-Ala: Indole-3-acetyl-L-alanine
ABA: Abscisic acid
GA_4_: Gibberellin acid 4
DAMs: Differentially accumulated metabolites
FM: Floral meristem
SAM: Shoot apical meristem
