# Supplementary material for: Integrated multi-omics and WGCNA analyses reveal pathways and candidate genes associated with branching flower development in Nymphaea prolifera: focusing on hormone homeostasis and flavonoid biosynthesis
Source: BMC Plant Biol. 2026 May 29;26:1280. doi: 10.1186/s12870-026-09140-2 (PMC13418433; doi:10.1186/s12870-026-09140-2)
Supplement: Supplementary file 1 — Supplementary Material 1. [file 12870_2026_9140_MOESM1_ESM.docx]

| 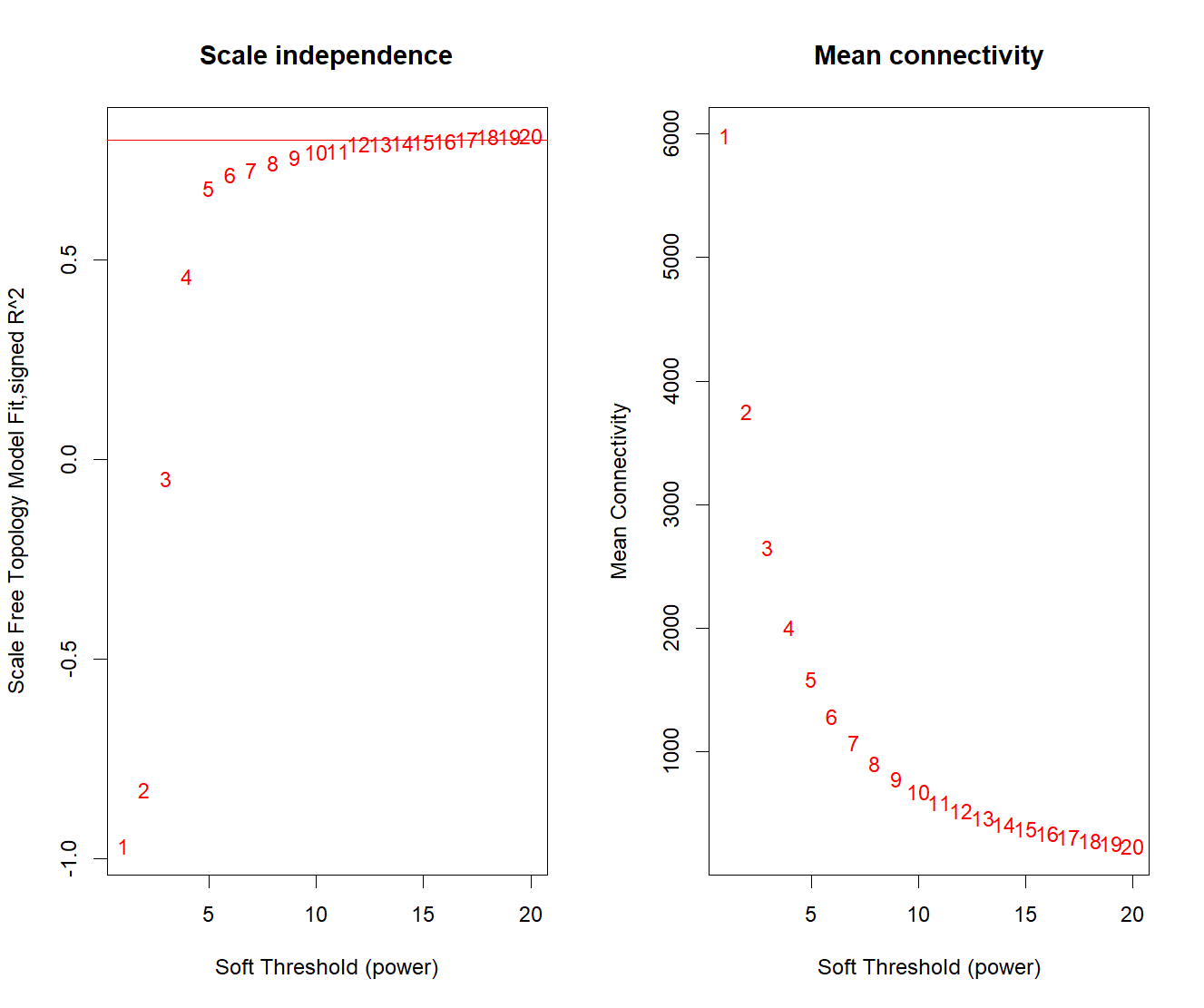 |
| --- |
| Sup Fig. 1 The decision of power value. Left figure, the horizontal axis represents different power values. The vertical axis represents the scale-free fit index as a function of the soft-thresholding power. The line represents that the correlation coefficient is equal to 0.80. Right figure, the average network connectivity under different power values. |

| 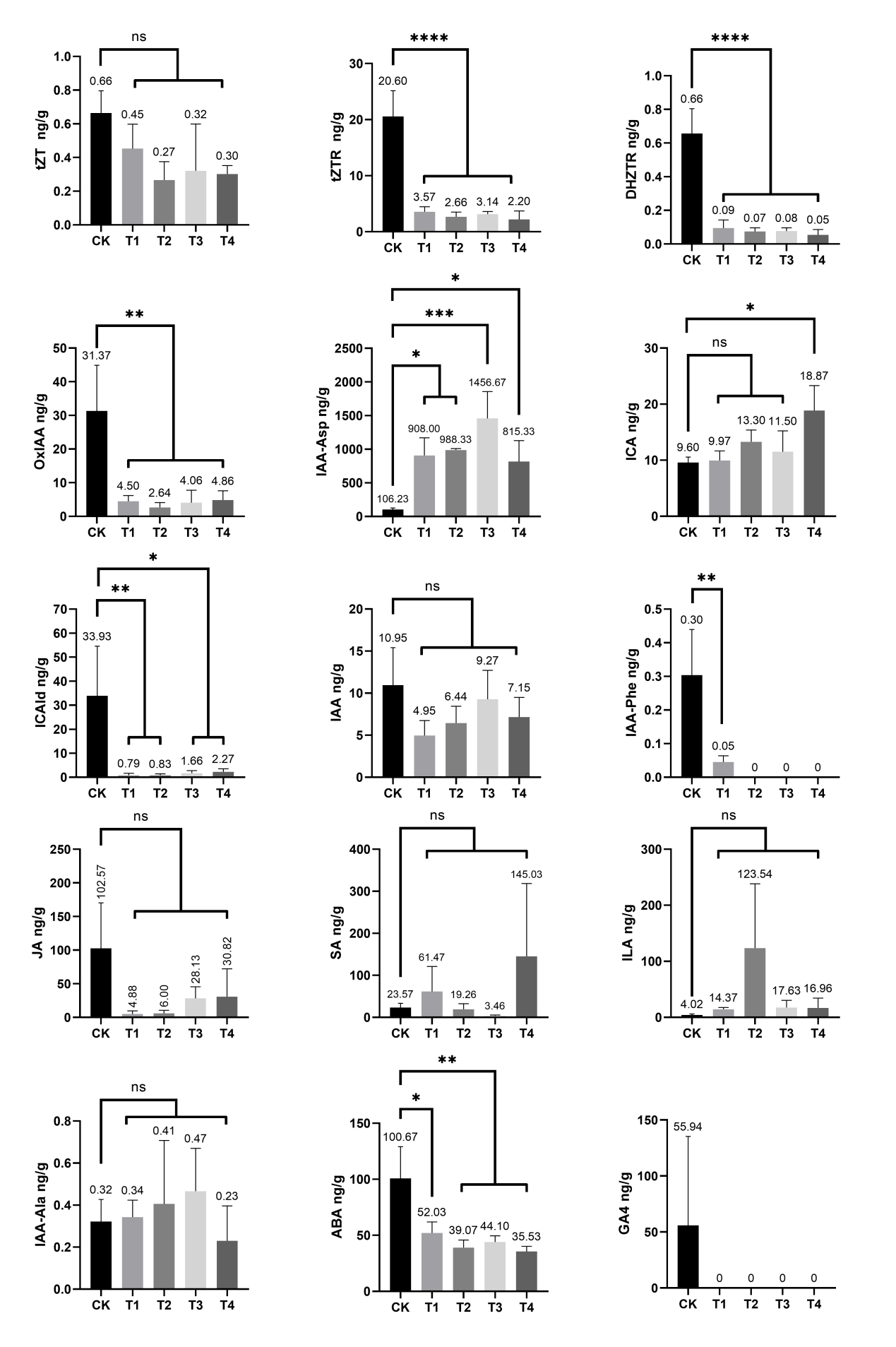 |
| --- |
| Sup Fig. 2 Histograms of 15 endogenous hormones in T1, T2, T3, T4, and CK samples. T test was used for analysis of variance. P-values are nominal. *P < 0.05, **P < 0.01, ***P < 0.001, ****P < 0.0001, 0 ng/g: substance was not detected. tZT: trans-zeatin. tZTR: trans-zeatin riboside. DHZTR: dihydrozeatin riboside. OxIAA: oxindole-3-acetic acid. IAA-Asp: indole-3-acetyl-L-aspartic acid. ICA: indole-3-carboxylic acid. ICAld: indole-3-carboxaldehyde. IAA: indole-3-acetic acid. IAA-Phe: indole-3-acetyl-L-phenylalanine. JA: jasmonic acid. SA: salicylic acid. ILA: indole-3-lactic acid. IAA-Ala: indole-3-acetyl-l-alanine. ABA: abscisic acid. GA_4_: gibberellin acid 4. |

| 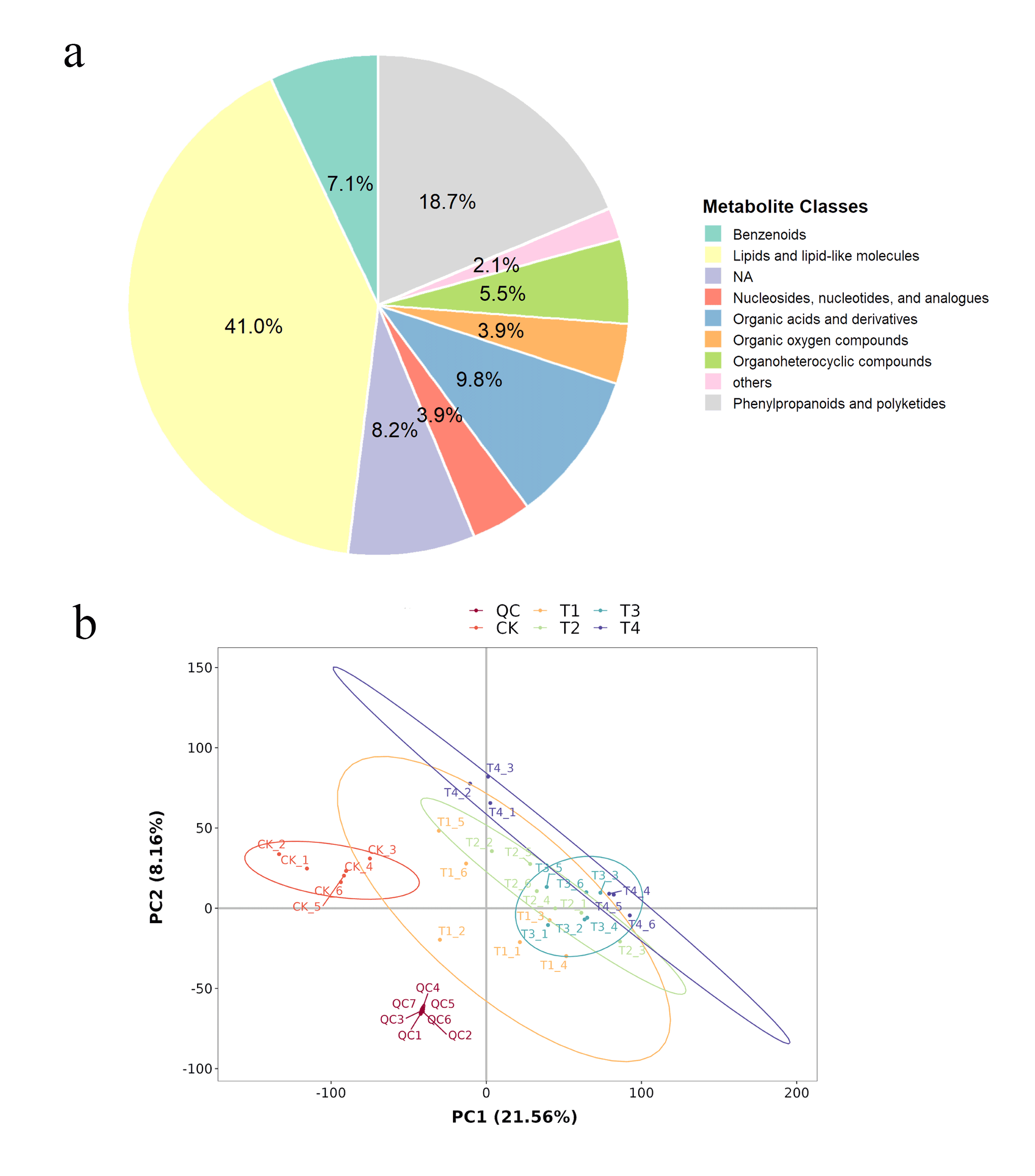 |
| --- |
| Sup Fig. 3 Classification of metabolites between groups from PLS-DA. a Classification and statistical analysis of the detected metabolites. b PLS-DA score plots for the samples from T1, T2, T3, T4, and CK. QC represents the quality control sample. |

| 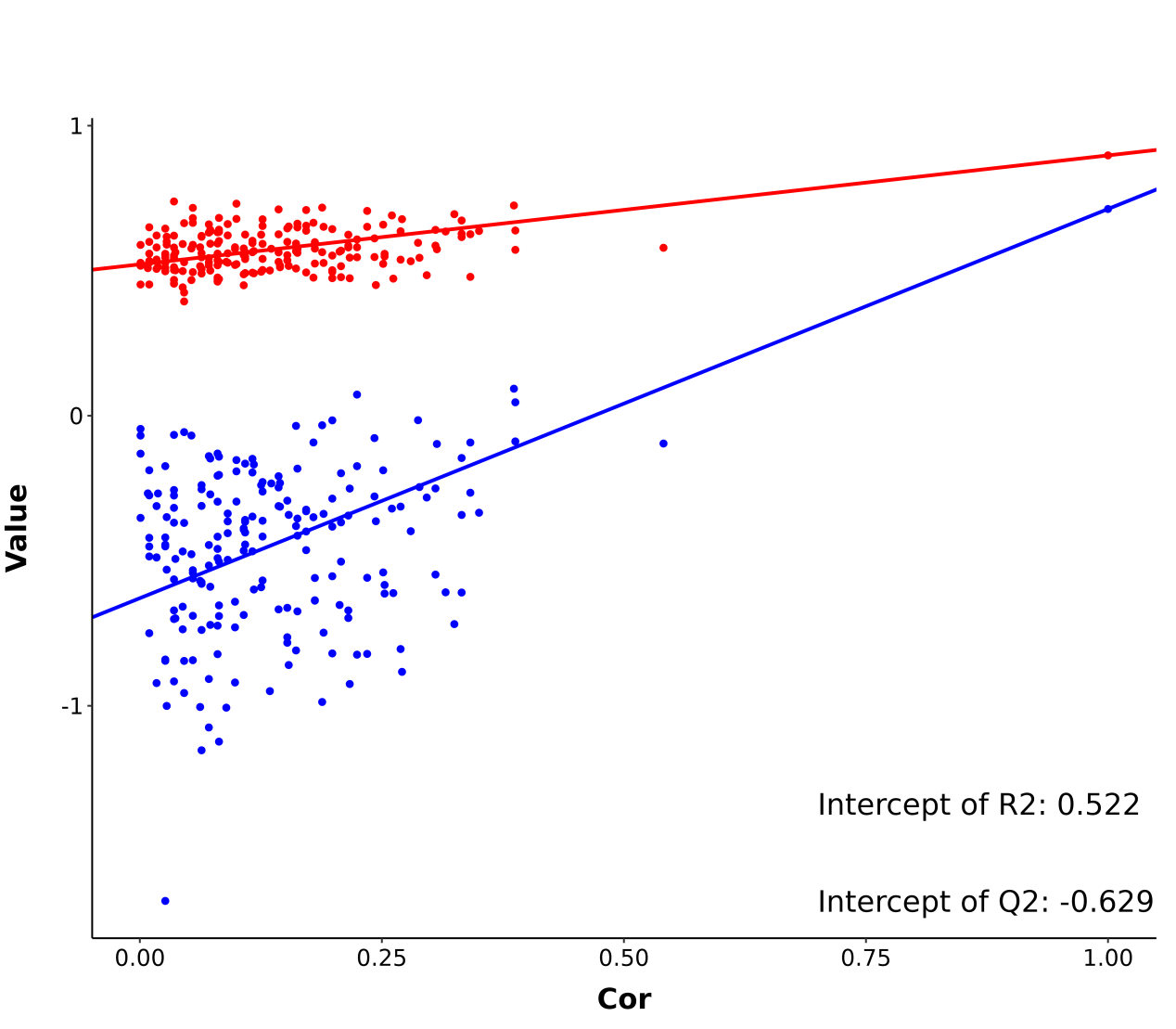 |
| --- |
| Sup Fig. 4 The replacement test plot |

| 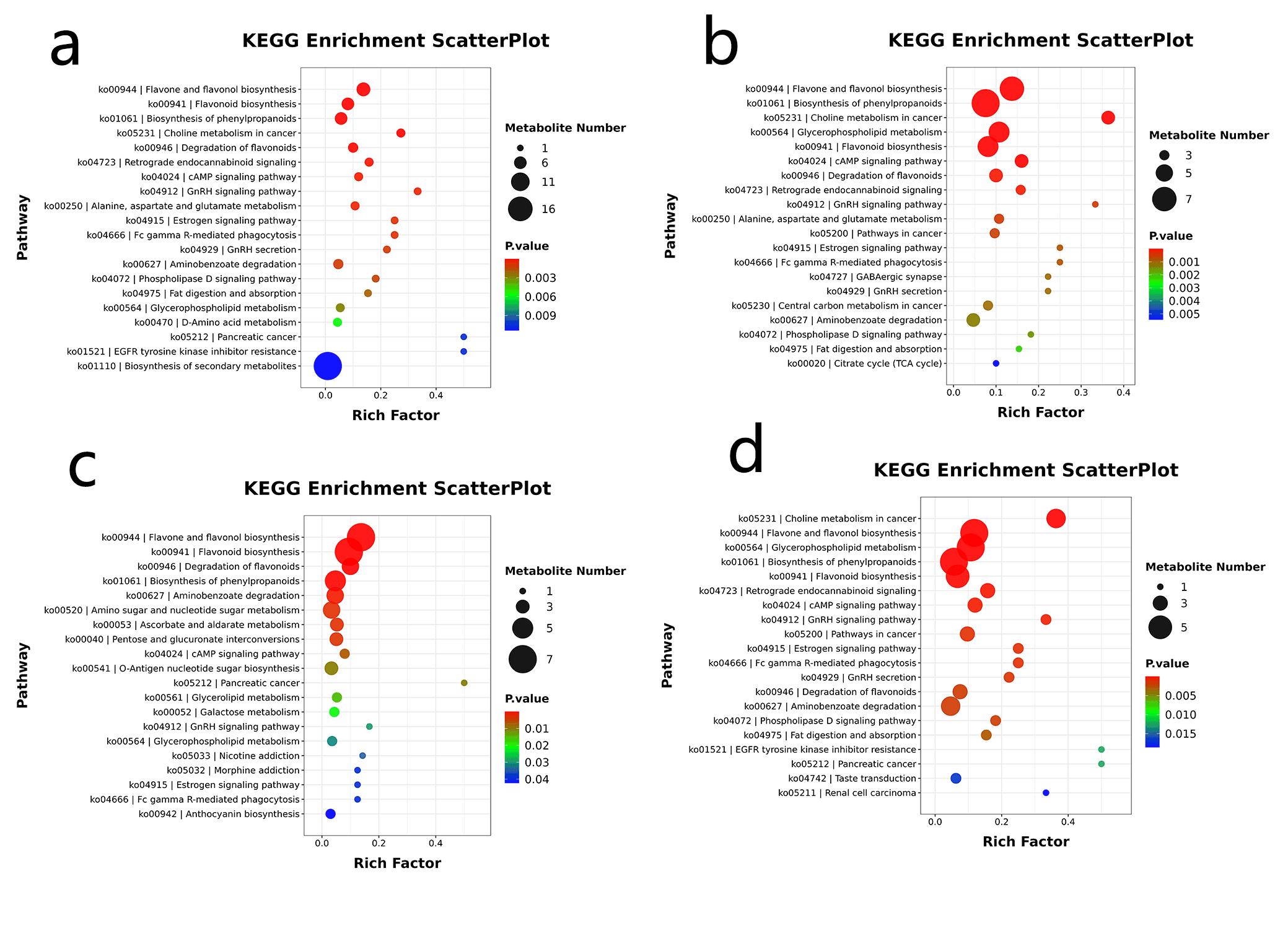 |
| --- |
| Sup Fig. 5 KEGG enrichment Bubble Diagram of four comparison groups. a KEGG enrichment anaylsis of DAMs in T1 vs CK. b KEGG enrichment anaylsis of DAMs in T2 vs CK. c KEGG enrichment anaylsis of DAMs in T3 vs CK. d KEGG enrichment anaylsis of DAMs in T4 vs CK. |

| 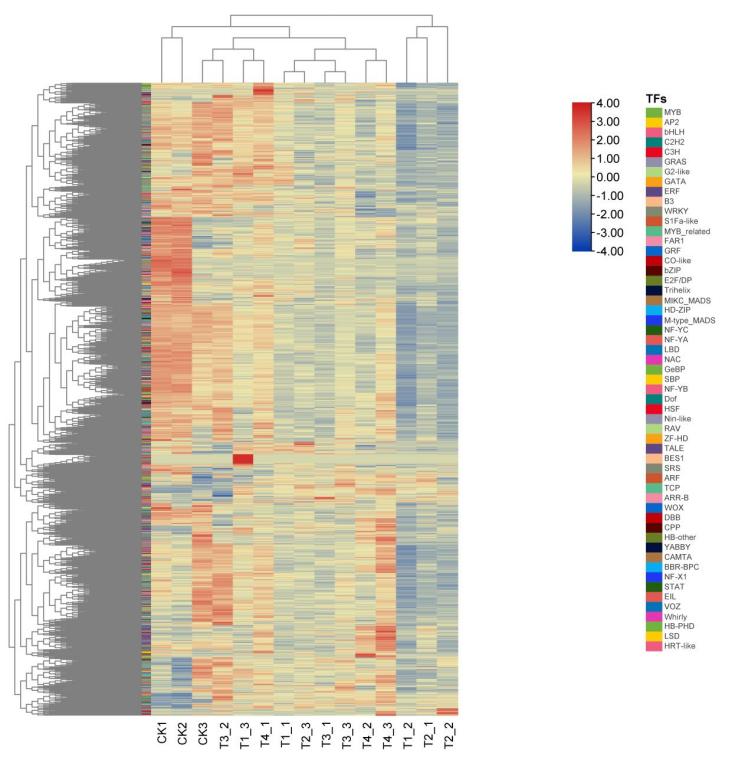 |
| --- |
| [Sup Fig. 6](Sup Fig (new).docx) Expression heat map of transcription factors (TFs) identified from the transcriptome data. Each row was log-scaled and normalized via the Normalized method. |

| 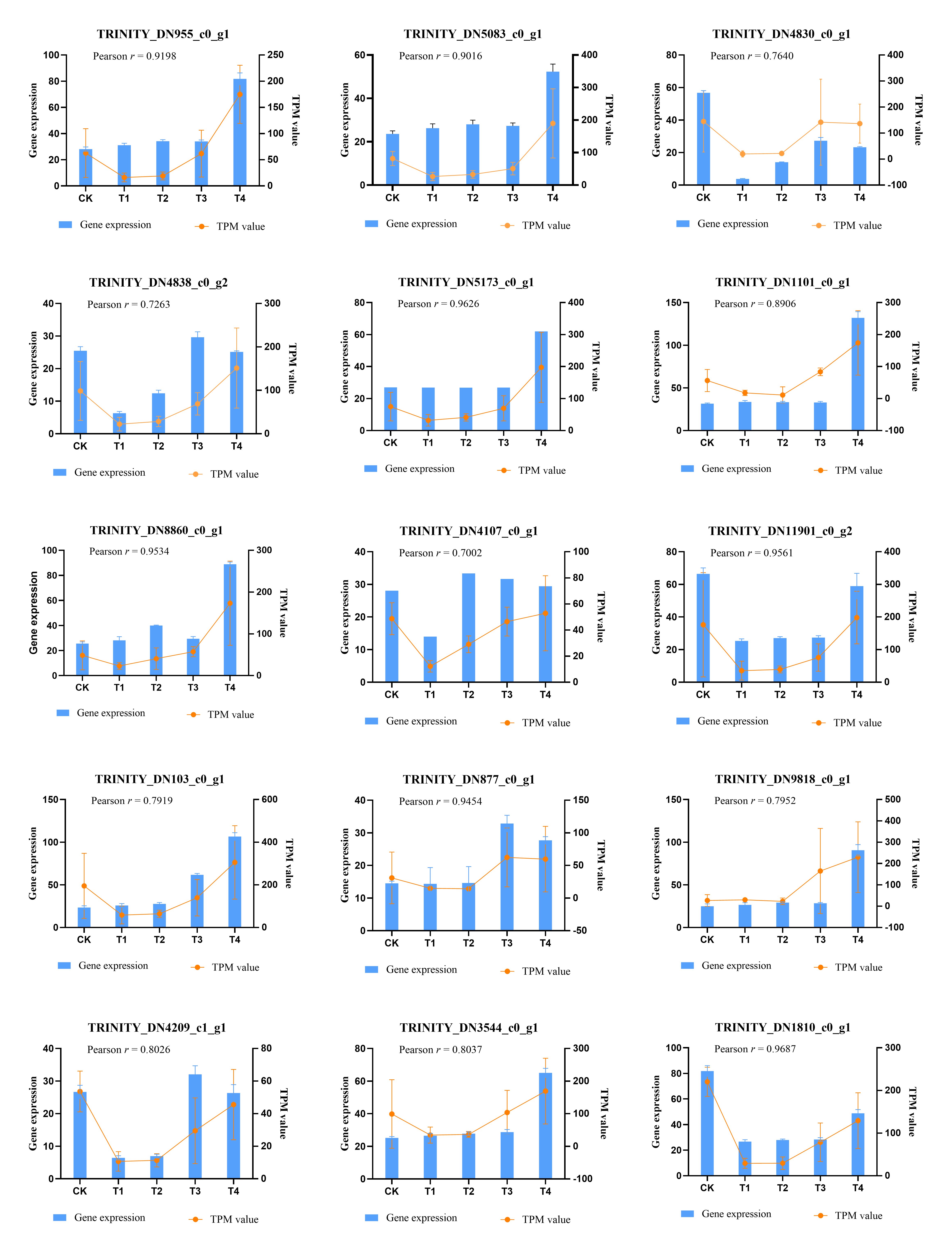 |
| --- |
| Sup Fig. 7 qRT-PCR validation of 15 genes. Line charts show the TPM values in RNA-seq data. Correlation analysis was performed with Pearsons correlation test. |

| 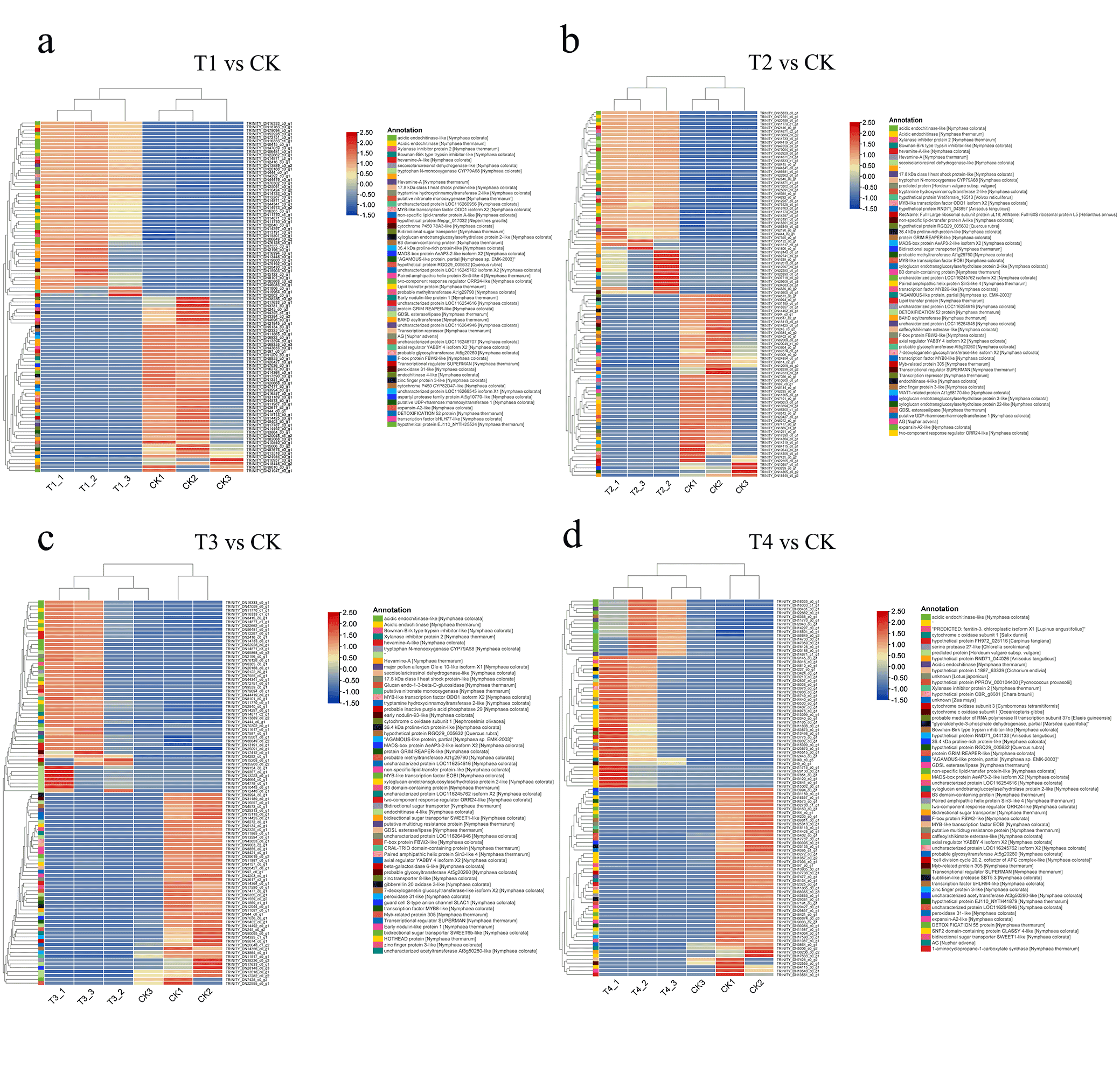 |
| --- |
| Sup Fig. 8 Heat maps of top 50 induced and top 50 suppressed genes.  a T1 vs CK. b T2 vs CK. c T3 vs CK. d T4 vs CK. Rows were log-transformed and scaled via the Normalized method. |

| 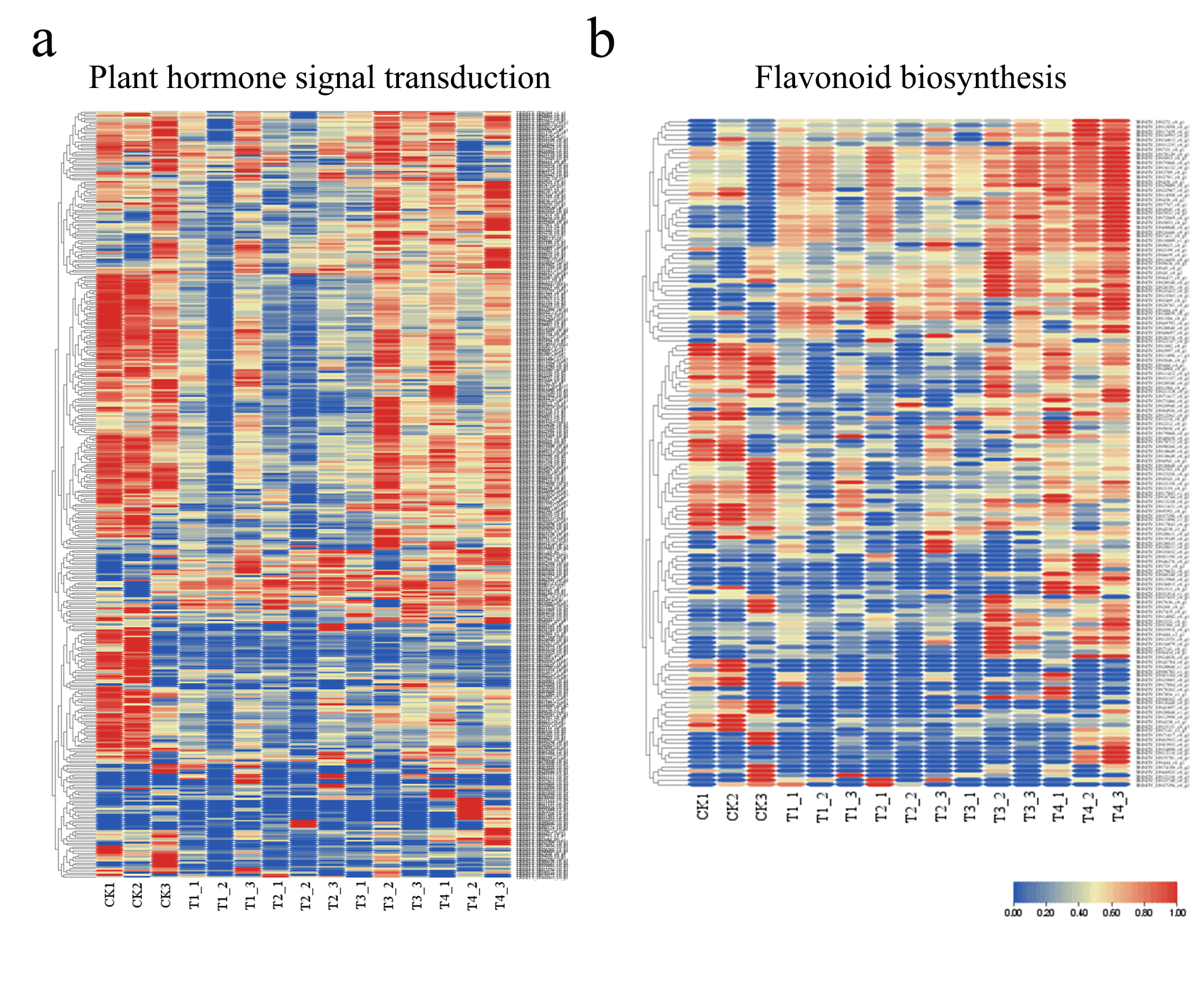 |
| --- |
| Sup Fig. 9 Expression heatmap of genes involved in plant hormone signal transduction (a) and flavonoid biosynthesis (b). |

| 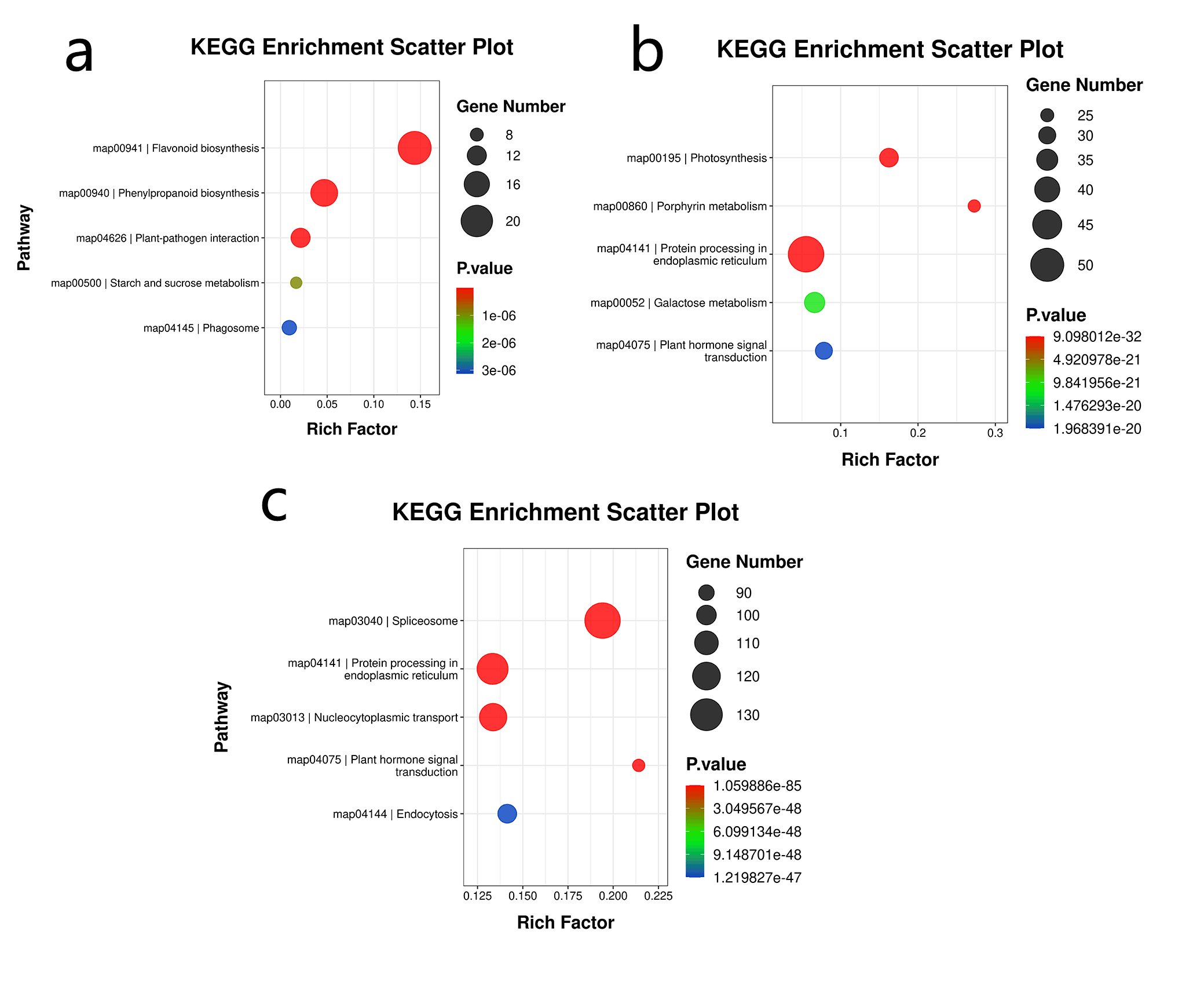 |
| --- |
| Sup Fig. 10 KEGG enrichment analysis of three modular genes scatter plot. a KEGG enrichment anaylsis of genes in Megreen module. b KEGG enrichment anaylsis of genes in MEblue module. c KEGG enrichment anaylsis of genes in MEturquoise module. |
