# Supplementary material for: Integrated multi-omics and WGCNA analyses reveal pathways and candidate genes associated with branching flower development in Nymphaea prolifera: focusing on hormone homeostasis and flavonoid biosynthesis
Source: BMC Plant Biol. 2026 May 29;26:1280. doi: 10.1186/s12870-026-09140-2 (PMC13418433; doi:10.1186/s12870-026-09140-2)
Supplement: Supplementary file 2 — Supplementary Material 2. [file 12870_2026_9140_MOESM2_ESM.docx]

Sup Tab. 1 Results of reference-based transcriptome sequencing against the *N. colorata genome*

| **Sample** | **Valid reads** | **Mapped reads** | **Unique Mapped reads** | **Multi Mapped reads** | **PE Mapped reads** | **Reads map to sense strand** | **Reads map to antisense strand** | **Non-splice reads** | **Splice reads** |
| --- | --- | --- | --- | --- | --- | --- | --- | --- | --- |
| CK1 | 36952170 | 1426490(3.86%) | 1369599(3.71%) | 56891(0.15%) | 254326(0.69%) | 688479(1.86%) | 681120(1.84%) | 551138(1.49%) | 818461(2.21%) |
| CK2 | 37197444 | 1469077(3.95%) | 1404642(3.78%) | 64435(0.17%) | 262726(0.71%) | 705922(1.90%) | 698720(1.88%) | 575775(1.55%) | 828867(2.23%) |
| CK3 | 42206250 | 1357047(3.22%) | 1284155(3.04%) | 72892(0.17%) | 233972(0.55%) | 645073(1.53%) | 639082(1.51%) | 528303(1.25%) | 755852(1.79%) |
| T1_1 | 38738514 | 1014096(2.62%) | 962252(2.48%) | 51844(0.13%) | 177376(0.46%) | 484430(1.25%) | 477822(1.23%) | 405787(1.05%) | 556465(1.44%) |
| T1_2 | 41113072 | 853751(2.08%) | 810635(1.97%) | 43116(0.10%) | 150516(0.37%) | 409312(1.00%) | 401323(0.98%) | 333858(0.81%) | 476777(1.16%) |
| T1_3 | 38355656 | 981213(2.56%) | 905275(2.36%) | 75938(0.20%) | 194094(0.51%) | 454304(1.18%) | 450971(1.18%) | 383717(1.00%) | 521558(1.36%) |
| T2_1 | 39789158 | 1065915(2.68%) | 989824(2.49%) | 76091(0.19%) | 216778(0.54%) | 501244(1.26%) | 488580(1.23%) | 410142(1.03%) | 579682(1.46%) |
| T2_2 | 36795326 | 811713(2.21%) | 711202(1.93%) | 100511(0.27%) | 201250(0.55%) | 360559(0.98%) | 350643(0.95%) | 312259(0.85%) | 398943(1.08%) |
| T2_3 | 33545044 | 934044(2.78%) | 876688(2.61%) | 57356(0.17%) | 177788(0.53%) | 442009(1.32%) | 434679(1.30%) | 368768(1.10%) | 507920(1.51%) |
| T3_1 | 36441566 | 988124(2.71%) | 915508(2.51%) | 72616(0.20%) | 198310(0.54%) | 463603(1.27%) | 451905(1.24%) | 386184(1.06%) | 529324(1.45%) |
| T3_2 | 42214344 | 1409061(3.34%) | 1361429(3.23%) | 47632(0.11%) | 222800(0.53%) | 680469(1.61%) | 680960(1.61%) | 580501(1.38%) | 780928(1.85%) |
| T3_3 | 37685024 | 1103768(2.93%) | 1055858(2.80%) | 47910(0.13%) | 186078(0.49%) | 532415(1.41%) | 523443(1.39%) | 443235(1.18%) | 612623(1.63%) |
| T4_1 | 34615766 | 1141166(3.30%) | 1054639(3.05%) | 86527(0.25%) | 235798(0.68%) | 531486(1.54%) | 523153(1.51%) | 456069(1.32%) | 598570(1.73%) |
| T4_2 | 41291416 | 1379608(3.34%) | 1220500(2.96%) | 159108(0.39%) | 338728(0.82%) | 616236(1.49%) | 604264(1.46%) | 542658(1.31%) | 677842(1.64%) |
| T4_3 | 43044260 | 1466197(3.41%) | 1357814(3.15%) | 108383(0.25%) | 293880(0.68%) | 683290(1.59%) | 674524(1.57%) | 596477(1.39%) | 761337(1.77%) |

Sup Tab. 2 Primer design of 15 genes

| gene ID | primer design |
| --- | --- |
| DN955_c0_g1-F | ACGAACGGACCACTCTGAACCT |
| DN955_c0_g1-R | CGTCGGCATACCAGTCGGATTC |
| DN5083_c0_g1-F | GGCGAACTCATCGTCTCTACCA |
| DN5083_c0_g1-R | TCCTTAGTTGCCGAGTCCTCAA |
| DN4830_c0_g1-F | CGCCAGCGGAGTTCCAATGT |
| DN4830_c0_g1-R | CCTTCCTTGCCATACTGCCTGA |
| DN4838_c0_g2-F | GGCAGGACCGATAACGCTATCA |
| DN4838_c0_g2-R | GCACAGAATACCGCCAGATGG |
| DN5173_c0_g1-F | CGACGCCTTCTACAACCTCCAA |
| DN5173_c0_g1-R | GCCGACTTCCTCAGCTTGTTCA |
| DN1101_c0_g1-F | CGTTTGCTGCTTGTGAGAGACA |
| DN1101_c0_g1-R | GCCGTTCCCAGTTGAGAATGC |
| DN8860_c0_g1-F | TGCCGTCCTCCTCCTCCTCTTA |
| DN8860_c0_g1-R | CGTACCGCTTCGACTCCGTTAG |
| DN4107_c0_g1-F | GCCATTCTCCCTCGCACCAAA |
| DN4107_c0_g1-R | CACCGCCACAGCATATCCACAA |
| DN11901_c0_g2-F | GCCTGCTTGCTTCGGATTCG |
| DN11901_c0_g2-R | CTCTTCCTCGTGGTCGGTTGTG |
| DN103_c0_g1-F | CACCGACTGCGAGAAGTTCCTC |
| DN103_c0_g1-R | GGTCACCACGAAGGCGAAGATG |
| DN877_c0_g1-F | GCTTGTGGTGTTCTTGCTTGGT |
| DN877_c0_g1-R | CCTGCCATCTGCTGCCTTCTT |
| DN9818_c0_g1-F | TGTCCGTCGCTTGCTCTCCT |
| DN9818_c0_g1-R | CTGCCATCCTCGAACTGCTTGA |
| DN4209_c1_g1-F | CGACTGCTGCTGGTGTTCTGA |
| DN4209_c1_g1-R | AGGCGATTGAATCCGACTCCAA |
| DN3544_c0_g1-F | TCGCGGCCTCAAGTACATCCA |
| DN3544_c0_g1-R | GCAAGCCCGAAGTCGCATATCT |
| DN1810_c0_g1 | GCTGGATCTGCATCTGCTGAGG |
| DN1810_c0_g1 | AGGAAGCCCATCTGCCAAACC |
| GADPH-F | CACGGCCACTGGAAGCA |
| GADPH-R | TCCTCAGGGTTCCTGATGCC |

Sup Tab. 3 Overview of sequencing quality control of 15 samples

| Sample | Raw_Reads | Raw_Bases | Valid_Reads | Valid_Bases | Valid% | Q20% | Q30% | GC% |
| --- | --- | --- | --- | --- | --- | --- | --- | --- |
| CK1 | 37899690 | 5.68G | 36811952 | 5.42G | 97.13 | 97.87 | 93.39 | 47.50 |
| CK2 | 37939514 | 5.69G | 37038994 | 5.47G | 97.63 | 98.30 | 94.64 | 47.49 |
| CK3 | 42953030 | 6.44G | 42142094 | 6.21G | 98.11 | 97.73 | 93.10 | 47.52 |
| T1_1 | 39563090 | 5.93G | 38892824 | 5.73G | 98.31 | 97.73 | 93.07 | 49.03 |
| T1_2 | 42187092 | 6.33G | 41436522 | 6.11G | 98.22 | 97.73 | 93.10 | 51.06 |
| T1_3 | 39081244 | 5.86G | 38422112 | 5.67G | 98.31 | 97.99 | 93.86 | 46.08 |
| T2_1 | 40719312 | 6.11G | 40001158 | 5.91G | 98.24 | 98.04 | 93.94 | 49.82 |
| T2_2 | 37718092 | 5.66G | 37006200 | 5.45G | 98.11 | 97.85 | 93.48 | 49.95 |
| T2_3 | 34340344 | 5.15G | 33671672 | 4.96G | 98.05 | 97.86 | 93.55 | 49.35 |
| T3_1 | 37221350 | 5.58G | 36579324 | 5.40G | 98.28 | 97.87 | 93.54 | 48.99 |
| T3_2 | 42973606 | 6.45G | 42170314 | 6.22G | 98.13 | 97.87 | 93.48 | 47.49 |
| T3_3 | 38413630 | 5.76G | 37704248 | 5.56G | 98.15 | 97.77 | 93.23 | 48.52 |
| T4_1 | 35276902 | 5.29G | 34650870 | 5.11G | 98.23 | 97.85 | 93.42 | 48.01 |
| T4_2 | 42174220 | 6.33G | 41363324 | 6.10G | 98.08 | 97.88 | 93.57 | 50.17 |
| T4_3 | 43810658 | 6.57G | 42985752 | 6.34G | 98.12 | 97.99 | 93.80 | 48.87 |

Sup Tab. 4 Overview of assembly results

| Index | All | GC% | Min Length | Median Length | Max Length | Total Assembled Bases | N50 |
| --- | --- | --- | --- | --- | --- | --- | --- |
| Transcript | 372069 | 42.52 | 177 | 1296 | 16822 | 652479612 | 2850 |
| Gene | 94503 | 42.63 | 201 | 417 | 16822 | 87690151 | 2035 |

Sup Tab. 5 Differentially expressed genes (DEGs) and their related information in four comparison groups.

Sup Tab 4a: [DEGs in T1vsCK](Supplementary Table 5/Supplementary Table 5a_T1_vs_CK_diff_exp.xlsx)

Sup Tab 4b: [DEGs in T2vsCK](Supplementary Table 5/Supplementary Table 5b_T2_vs_CK_diff_exp.xlsx)

Sup Tab 4c: [DEGs in T3vsCK](Supplementary Table 5/Supplementary Table 5c_T3_vs_CK_diff_exp.xlsx)

Sup Tab 4d: [DEGs in T4vsCK](Supplementary Table 5/Supplementary Table 5d_T4_vs_CK_diff_exp.xlsx)

Note：This table is compiled in Excel format. Since it cannot be directly presented in the Word document, it will be provided via a dedicated link.

Sup Tab. 6 KEGG enrichment analysis of four comparison groups

| Group | PathwayEntry | PathwayDefinition | S gene number | TS gene number | B gene number | TB gene number | pvalue | Rich factor |
| --- | --- | --- | --- | --- | --- | --- | --- | --- |
| T1vsCK | map00520 | Amino sugar and nucleotide sugar metabolism | 38 | 509 | 637 | 15609 | 0.00 | 0.546636387 |
| T1vsCK | map04075 | Plant hormone signal transduction | 28 | 509 | 383 | 15609 | 0.00 | 0.446049898 |
| T1vsCK | map00500 | Starch and sucrose metabolism | 34 | 509 | 416 | 15609 | 0.00 | 0.398985502 |
| T1vsCK | map02010 | ABC transporters | 20 | 509 | 193 | 15609 | 0.00 | 0.314680633 |
| T1vsCK | map00940 | Phenylpropanoid biosynthesis | 50 | 509 | 363 | 15609 | 0.00 | 0.236744186 |
| T1vsCK | map00561 | Glycerolipid metabolism | 28 | 509 | 201 | 15609 | 0.00 | 0.23408885 |
| T1vsCK | map00945 | Stilbenoid, diarylheptanoid and gingerol biosynthesis | 17 | 509 | 105 | 15609 | 0.00 | 0.201410951 |
| T1vsCK | map00904 | Diterpenoid biosynthesis | 13 | 509 | 74 | 15609 | 0.00 | 0.185622693 |
| T1vsCK | map00941 | Flavonoid biosynthesis | 31 | 509 | 146 | 15609 | 0 | 0.153579717 |
| T1vsCK | map00591 | Linoleic acid metabolism | 5 | 509 | 21 | 15609 | 0.00 | 0.136959446 |
| T2vsCK | map04075 | Plant hormone signal transduction | 43 | 965 | 383 | 15609 | 0.00 | 0.550658758 |
| T2vsCK | map00500 | Starch and sucrose metabolism | 53 | 965 | 416 | 15609 | 0.00 | 0.485254637 |
| T2vsCK | map00195 | Photosynthesis | 27 | 965 | 197 | 15609 | 0.00 | 0.451081166 |
| T2vsCK | map00561 | Glycerolipid metabolism | 32 | 965 | 201 | 15609 | 0.00 | 0.388327648 |
| T2vsCK | map00460 | Cyanoamino acid metabolism | 19 | 965 | 118 | 15609 | 0.00 | 0.383955275 |
| T2vsCK | map00945 | Stilbenoid, diarylheptanoid and gingerol biosynthesis | 17 | 965 | 105 | 15609 | 0.00 | 0.381849838 |
| T2vsCK | map04712 | Circadian rhythm - plant | 18 | 965 | 100 | 15609 | 0.00 | 0.343462817 |
| T2vsCK | map00940 | Phenylpropanoid biosynthesis | 66 | 965 | 363 | 15609 | 0.00 | 0.340028189 |
| T2vsCK | map00904 | Diterpenoid biosynthesis | 14 | 965 | 74 | 15609 | 0.00 | 0.326780337 |
| T2vsCK | map00941 | Flavonoid biosynthesis | 39 | 965 | 146 | 15609 | 0.00 | 0.231441098 |
| T3vsCK | map04075 | Plant hormone signal transduction | 27 | 571 | 383 | 15609 | 0.00 | 0.518914776 |
| T3vsCK | map00196 | Photosynthesis - antenna proteins | 16 | 571 | 168 | 15609 | 0.00 | 0.384105324 |
| T3vsCK | map04712 | Circadian rhythm - plant | 10 | 571 | 100 | 15609 | 0.00 | 0.365814594 |
| T3vsCK | map00860 | Porphyrin and chlorophyll metabolism | 9 | 571 | 88 | 15609 | 0.00 | 0.357685381 |
| T3vsCK | map00130 | Ubiquinone and other terpenoid-quinone biosynthesis | 8 | 571 | 75 | 15609 | 0.01 | 0.342951182 |
| T3vsCK | map00940 | Phenylpropanoid biosynthesis | 45 | 571 | 363 | 15609 | 0.00 | 0.295090439 |
| T3vsCK | map00561 | Glycerolipid metabolism | 27 | 571 | 201 | 15609 | 0.00 | 0.272328642 |
| T3vsCK | map00945 | Stilbenoid, diarylheptanoid and gingerol biosynthesis | 18 | 571 | 105 | 15609 | 0.00 | 0.213391847 |
| T3vsCK | map00908 | Zeatin biosynthesis | 6 | 571 | 35 | 15609 | 0.00 | 0.213391847 |
| T3vsCK | map00941 | Flavonoid biosynthesis | 32 | 571 | 146 | 15609 | 0 | 0.166902909 |
| T4vsCK | map04145 | Phagosome | 75 | 854 | 957 | 15609 | 0.00 | 0.69812544 |
| T4vsCK | map00561 | Glycerolipid metabolism | 24 | 854 | 201 | 15609 | 0.00 | 0.45821321 |
| T4vsCK | map00073 | Cutin, suberine and wax biosynthesis | 10 | 854 | 75 | 15609 | 0.01 | 0.410340188 |
| T4vsCK | map00940 | Phenylpropanoid biosynthesis | 74 | 854 | 363 | 15609 | 0 | 0.268384664 |
| T4vsCK | map00901 | Indole alkaloid biosynthesis | 4 | 854 | 16 | 15609 | 0.01 | 0.2188481 |
| T4vsCK | map00945 | Stilbenoid, diarylheptanoid and gingerol biosynthesis | 29 | 854 | 105 | 15609 | 0.00 | 0.198095263 |
| T4vsCK | map00941 | Flavonoid biosynthesis | 46 | 854 | 146 | 15609 | 0 | 0.17365121 |
| T4vsCK | map00944 | Flavone and flavonol biosynthesis | 8 | 854 | 25 | 15609 | 0.00 | 0.170975078 |

Note: The table shows the top 10 pathways with the lowest enrichment significance in each comparison group.

Sup Tab. 7 14 flavonoid structural genes associated with flavonoid DAMs in the MEgreen module

| gene_id | gene_name | module |
| --- | --- | --- |
| TRINITY_DN3931_c0_g2 | DFR | green |
| TRINITY_DN60040_c0_g1 | - | green |
| TRINITY_DN75066_c0_g3 | PKS5 | green |
| TRINITY_DN7411_c0_g1 | CYP75A1 | green |
| TRINITY_DN4945_c0_g1 | CHI1 | green |
| TRINITY_DN438_c0_g1 | AN3 | green |
| TRINITY_DN18009_c1_g1 | - | green |
| TRINITY_DN2781_c0_g1 | ANR | green |
| TRINITY_DN16446_c0_g1 | CYP75A6 | green |
| TRINITY_DN725_c0_g1 | CHS-1B | green |
| TRINITY_DN16132_c0_g1 | ANR | green |
| TRINITY_DN3789_c0_g1 | CHIL2 | green |
| TRINITY_DN78149_c0_g1 | CHS3 | green |
| TRINITY_DN5386_c0_g1 | DFR | green |
